# X-ray-activated long persistent phosphors featuring strong UVC afterglow emissions

**DOI:** 10.1038/s41377-018-0089-7

**Published:** 2018-11-14

**Authors:** Yan-Min Yang, Zhi-Yong Li, Jun-Ying Zhang, Yue Lu, Shao-Qiang Guo, Qing Zhao, Xin Wang, Zi-Jun Yong, Hong Li, Ju-Ping Ma, Yoshihiro Kuroiwa, Chikako Moriyoshi, Li-Li Hu, Li-Yan Zhang, Li-Rong Zheng, Hong-Tao Sun

**Affiliations:** 1 College of Physics Science and Technology, Hebei University, 071002 Baoding, China; 2 College of Chemistry, Chemical Engineering and Materials Science, Soochow University, 215123 Jiangsu, China; 3 Department of Physics, Beihang University, 100191 Beijing, China; 4 Department of Physical Science, Hiroshima University, Higashihiroshima, Hiroshima, 739-8526 Japan; 5 Shanghai Institute of Optics and Fine Mechanics, Chinese Academy of Sciences, 201800 Shanghai, China; 6 Beijing Synchrotron Radiation Facility, Institute of High Energy Physics, Chinese Academy of Sciences, 100049 Beijing, China

## Abstract

Phosphors emitting visible and near-infrared persistent luminescence have been explored extensively owing to their unusual properties and commercial interest in their applications such as glow-in-the-dark paints, optical information storage, and in vivo bioimaging. However, no persistent phosphor that features emissions in the ultraviolet C range (200–280 nm) has been known to exist so far. Here, we demonstrate a strategy for creating a new generation of persistent phosphor that exhibits strong ultraviolet C emission with an initial power density over 10 milliwatts per square meter and an afterglow of more than 2 h. Experimental characterizations coupled with first-principles calculations have revealed that structural defects associated with oxygen introduction-induced anion vacancies in fluoride elpasolite can function as electron traps, which capture and store a large number of electrons triggered by X-ray irradiation. Notably, we show that the ultraviolet C afterglow intensity of the yielded phosphor is sufficiently strong for sterilization. Our discovery of this ultraviolet C afterglow opens up new avenues for research on persistent phosphors, and it offers new perspectives on their applications in terms of sterilization, disinfection, drug release, cancer treatment, anti-counterfeiting, and beyond.

## Phosphors: Persistent luminescence of UVC light

A new category of the luminescent compounds called phosphors displays strong and persistent emission of UVC light, with potential applications including disinfection, drug-release, cancer treatment, and anti-counterfeiting measures. Researchers in China and Japan, led by Hong-Tao Sun at Soochow University in China, created several examples of the new generation of phosphors, which are stimulated into luminescence by X-ray irradiation. The bright UVC emission persists for more than two hours. Theoretical and experimental results suggest generally applicable structural explanations for the luminescent properties. The researchers believe this is the first discovery of persistent phosphors luminescing in the germicidal UVC range. The emitted light is sufficiently strong for sterilization applications. The ability to further stimulate the phosphors with near-infrared light, which can pass through biological tissues, suggests potential for treating cancer or triggering drug release.

## Introduction

Persistent luminescence is an optical phenomenon in which a material stores excitation energy in excited states and the resulting luminescence lasts for an appreciable time after the excitation has stopped^1,2^. Phosphors exhibiting persistent luminescence have received significant attention and have been commercialized for a wide range of applications^3–12^. Since the pioneering work of Matsuzawa et al. ^13^ blue persistent CaAl_2_O_4_:Eu^2+^, Nd^3+^ (ref. ^14^), green persistent SrAl_2_O_4_:Eu^2+^, Dy^3+^ (ref. ^13^), and red persistent Y_2_O_2_S:Eu^3+^, Mg^2+^, and Ti^4+^ (ref. ^15^) have been developed. Recent efforts have resulted in the creation of a series of near-infrared (NIR) persistent phosphors, such as Cr^3+^-doped zinc gallogermanates that can be employed for the in vivo biological imaging and the in vitro targeting of cancerous cells^16–26^. Despite these significant achievements, most of the persistent phosphors reported thus far luminesce in the visible and NIR spectral regions^27^, and no persistent phosphors exhibiting ultraviolet C (UVC) luminescence are known to exist. It is well known that UVC light in the 200–280 nm range is germicidal, i.e., it can kill bacteria, viruses, and other pathogens by destroying nucleic acids and disabling their ability to multiply^28^. However, the design and synthesis of such a long-lasting phosphor has not been possible thus far.

Two classes of centers, emitters and traps, are required in persistent phosphors. Emitters are optical centers that luminesce after being excited, whereas traps, which are usually associated with defects in host lattices, store excitation energy and then release it slowly to the emitters by virtue of thermal stimulations^2^. Since the emitter determines the emission wavelength, the first required condition for the design of UVC-persistent phosphors is the choice of a suitable emitter that can luminesce in the UVC. Rare earth (RE) ions have been widely adopted as emitters in persistent phosphors owing to their intraconfigurational transitions. It is noted that some RE ions (e.g., Pr^3+^) possess strong, high-energy, interconfigurational transitions, which thus could be employed as candidate emitters in UVC persistent phosphors^29–31^. The second required condition is the selection of the candidate host materials with a large bandgap and appropriate traps. In this respect, fluoride crystalline lattices can be considered because of their relatively large band gaps and the easy creation of anionic defects^32^. The third condition is that the material system can be efficiently activated (i.e., charged) by suitable excitation sources. Given the large bandgap required for the host, X-ray irradiation may be appropriate. Another requirement relates to the persistent time and intensity of the UVC emission, which should be as long as possible and strong enough to satisfy some practical applications.

Here, we demonstrate a strategy for the creation of a new generation of persistent phosphor featuring UVC emission (which we refer to as a UVC persistent phosphor) by judiciously selecting defect-bearing fluoride elpasolite (i.e., Cs_2_NaYF_6_) as a host and Pr^3+^ ions as emitters. The resulting phosphors show bright UVC emissions that can last over 2 h after X-ray irradiation. To our knowledge, our work is the first discovery of persistent phosphors capable of luminescing in the UVC. A broad range of experimental characterizations combined with first-principles calculations suggest that oxygen introduction-induced fluorine vacancies act as electron traps, enabling the system to capture a large number of electrons upon X-ray irradiation. This results in strong UVC persistent luminescence corresponding to the 4*f*5*d*–4*f*^2^ transition of Pr^3+^ when releasing trapped electrons. We show that the UVC persistent luminescence of this phosphor is strong enough to be used for sterilization. Importantly, the phosphors can be charged repeatedly by X-ray or poststimulated by NIR light in the first biological window, thus offering an attractive prospect for applications, such as the in vivo killing of pathogens and cancer cells. This work offers a protocol for the design and preparation of UVC persistent phosphors and opens up new avenues for a wide variety of practical applications.

## Results

**Fig. 1:**
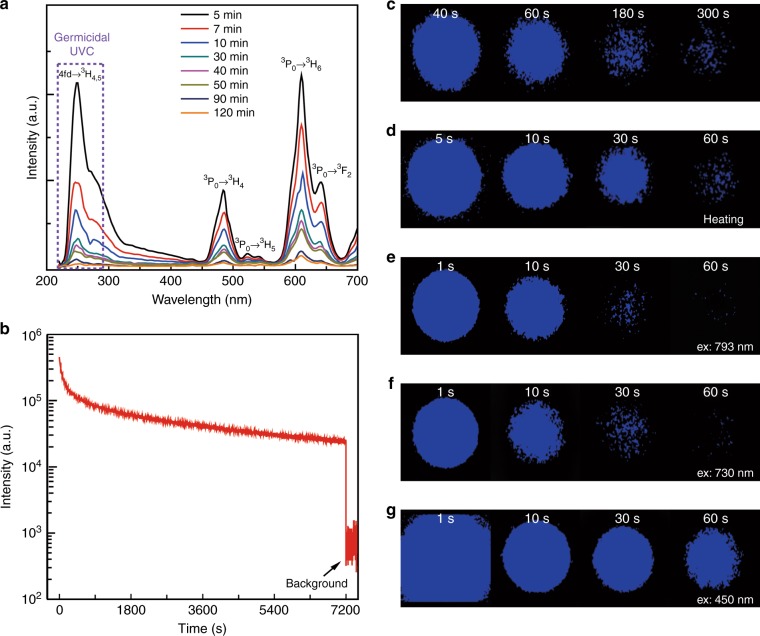
UVC afterglow of Pr^3+^-doped fluoride elpasolite with a nominal composition of Cs_2_NaY_0.99_F_6_:0.01Pr^3+^ and X-ray irradiation for 1000 s. **a** The afterglow spectra recorded at different times after ceasing X-ray irradiation. The emission band peaking at 250 nm and the emission shoulder at 270 nm can be assigned to the transitions of 4*f*5*d* → ^3^H_4_ and 4*f*5*d* → ^3^H_5_, respectively. In addition to the UVC emissions, visible emission bands were also observed. **b** Afterglow decay detected at 250 nm as a function of time. The data were taken from 5 min after stopping the X-ray irradiation. **c** UVC images of phosphors taken at different afterglow times; the images after 300 s are shown in Supplementary Fig. 10a. **d** UVC images of the 24-h-decayed phosphors heated to 200 °C on a hot plate. **e**–**g** UVC images of the 24-h-decayed phosphors under laser irradiation with different wavelengths of (**e**) 793 nm, (**f**) 730 nm, and (**g**) 450 nm. The excitation power density is 1.77 W/cm^2^ for the 793, 730, and 450 nm excitation wavelengths

We chose elpasolite Cs_2_NaYF_6_ as the host and Pr^3+^ ions as emitters with the ideas that (1) the Cs element has a strong X-ray absorption capability that makes the system capable of charging by X-ray irradiation, (2) Cs_2_NaYF_6_ has a large bandgap and a propensity for the formation of structural defects that are likely to act as electron traps^32^, and (3) the 4*f*5*d*–4*f*^2^ transition of Pr^3+^ ions can result in UVC emission^29–31^. The micrometer-sized phosphor with a nominal composition of Cs_2_NaY_(1*−x*)_F_6_:*x*Pr^3+^ was synthesized through a solid-state reaction method (Supplementary Fig. 1). The major reflections of X-ray diffraction (XRD) patterns for the products can be indexed with an Fm-3m space group that corresponds to the cubic elpasolite (Supplementary Fig. 2), consistent with JCPDS no. 74-0043. In this double perovskite structure, both Y and Na coordinate with six fluorine atoms, and doped Pr^3+^ ions are expected to substitute for Y^3+^ ions (Supplementary Fig. 3). We first characterized the photoluminescence properties of the yielded product. Under 288 nm excitation, the product shows luminescence bands at 486 and 610 nm, which can be assigned to the ^3^P_0_ → ^3^H_4_ and ^3^P_0_ → ^3^H_6_ transitions of Pr^3+^, respectively^33^ (Supplementary Fig. 4). We note that after photo-excitation ceases, the photoluminescence quickly disappears, indicating that excitation at 288 nm cannot result in the charging of this system, which is a necessity for long persistent luminescence. Interestingly, we find that after X-ray irradiation, the sample displayed strong, long-lasting UVC persistent luminescence peaking at ~250 nm owing to the 4*f*5*d* → ^3^H_4_ electronic transition of Pr^3+^, accompanied by visible bands of 4*f*^2^–4*f*^2^ intraconfigurational transitions (Fig. 1a). Figure 1b displays the afterglow decay of Cs_2_NaY_0.99_F_6_:0.01Pr^3+^ detected at 250 nm following irradiation with an X-ray source for 1000 s that corresponds to a dose of 20 Gy (Supplementary Fig. 5). We note that all data regarding the afterglow intensity versus time were taken using a spectrofluorometer from 5 min after stopping the X-ray irradiation, to avoid the measurement artifact owing to fast early decay. Clearly, the persistent luminescence of Cs_2_NaY_0.99_F_6_:0.01Pr^3+^ can last over 2 h, and after 2 h, the intensity is still over one order of magnitude stronger than the background signal of the detection system (Fig. 1b). Additionally, we find that the afterglow behavior is intimately associated with the concentration of Pr^3+^ ions and the X-ray irradiation duration. The optimal concentration is determined to be *x* = 0.01 (Supplementary Fig. 6), and 1000 s of X-ray irradiation of Cs_2_NaY_0.99_F_6_:0.01Pr^3+^ gives rise to the strongest UVC persistent luminescence (Supplementary Fig. 7). The afterglow spectra recorded at different times reveal that the lineshape of the luminescence changes with times; the UVC emission decays faster than the visible emissions (Fig. 1b, Supplementary Fig. 8). In addition to the emission bands mentioned above, we note that an emission shoulder at ~340 nm in the afterglow spectra occurs, which is confirmed to be from the host material (Supplementary Fig. 9).

We further confirmed the UVC emission by using a UVC imager. Figure 1c and Supplementary Fig. 10a display the dependence of the UVC emission intensity on the decay time for Cs_2_NaY_0.99_F_6_:0.01Pr^3+^ irradiated by the X-ray for 1000 s. Clearly, the decay decreases much faster during the first several minutes, and then it occurs slowly (Supplementary Fig. 10b). The initial afterglow UVC emission intensity of Cs_2_NaY_0.99_F_6_:0.01Pr^3+^ after stopping the X-ray irradiation was estimated to be over 10 milliwatts per square meter (Materials and methods). Heating the 24-h-decayed phosphor at 200 °C gave rise to strong UVC luminescence again, with a lasting time of over 5 min (Fig. 1d, Supplementary Fig. 10c). Interestingly, we find that these trapped electrons can also be liberated by laser irradiation with various photon energies (Fig. 1e–g, Supplementary Fig. 11). Specifically, irradiation of 450 nm light causes stronger emissions than those of 730 and 793 nm light, suggesting that there are more residual trapped electrons located in deeper traps. Collectively, these observations provide an indication that the UVC persistent phosphors developed here feature a diversity of traps, and that a large number of residual electrons is located in deep traps after the room temperature release of electrons in shallow ones. We underscore that this characteristic is extremely attractive and of vital importance for some applications of these UVC phosphors. For instance, although a relatively long-time X-ray irradiation is required to charge the system at present, the release of stored electrons partly in the form of UVC photons under NIR-light stimuli renders it attractive for the in vivo killing of pathogens and cancer cells.

**Fig. 2:**
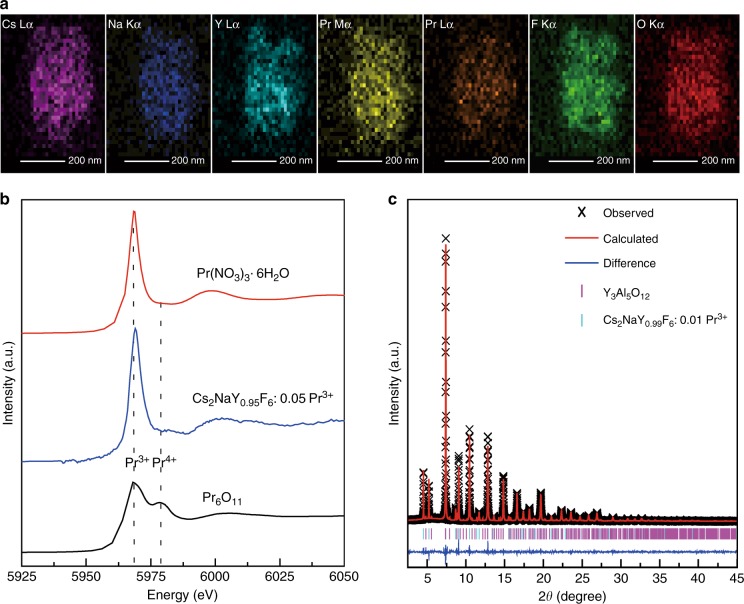
Element distribution, oxidation states of Pr, and structural characterization by synchrotron XRD. **a** EDS elemental mappings of Cs_2_NaY_0.99_F_6_:0.01Pr^3+^. **b** Pr L_III_-edge XANES spectra of as-synthesized Cs_2_NaY_0.95_F_6_:0.05Pr^3+^, Pr(NO_3_)_3_·6H_2_O and Pr_6_O_11_. **c** Rietveld refinement against the high-resolution synchrotron XRD pattern of Cs_2_NaY_0.99_F_6_:0.01Pr^3+^. Due to the presence of a small amount of YAG (JCPDS no. 72-1315), a two-phase model is used for the Rietveld refinement

The above results clearly show that Pr^3+^ ions act as emitters, but the identity of the traps remains unclear in this persistent phosphor. To understand the mechanism of persistent luminescence observed here, we next performed detailed experimental characterizations of the composition, structure, and possible defects in this system using a wide range of techniques. The composition of the yielded powders was first characterized by transmission electron microscopy and energy-dispersive X-ray spectroscopy (TEM-EDS). Interestingly, in addition to the presence of the expected constituent elements of Cs_2_NaY_0.99_F_6_:0.01Pr^3+^, we find that the oxygen element is nearly homogeneously distributed in the particle (Fig. 2a). This elemental distribution was also verified by the scanning electron microscopy (SEM)-EDS measurements (Supplementary Fig. 12), yielding an average O/F molar ratio of 12.3%. To ascertain that the oxygen distribution is not limited to the surface region, we also performed the X-ray photoelectron spectroscopy (XPS) measurements. As illustrated in Supplementary Fig. 13, the phosphor has a significant amount of oxygen, which is observed even after argon plasma etching to remove the outer surface layer. This suggests that the oxygen atoms extend throughout the bulk of the material instead of merely on the surface. We surmise that this distribution may be caused by the insufficient fluorination of oxide precursors by NH_4_F during synthesis. Additionally, given that Pr ions are expected to play a role in the energy harvest and release within this UVC persistent phosphor, we next probed the oxidation state of Pr ions by Pr L_III_-edge X-ray absorption near-edge structure (XANES). Two typical compounds containing Pr, Pr(NO_3_)_3_·6H_2_O, and Pr_6_O_11_, were employed as reference samples. As displayed in Fig. 2b, the XANES spectra signify the absence of Pr^4+^ ions in the as-synthesized phosphor, suggesting the isovalent substitution of Pr^3+^ for Y^3+^ ions.

To obtain more information concerning the structure of the phosphor, we performed a high-resolution synchrotron XRD measurement. Most of the diffraction pattern is readily indexed with an Fm−3m space group, and some weak extra diffraction lines assigned to yttrium aluminum garnet (YAG) were detected. The occurrence of YAG in the product results from the reaction between the precursors and the corundum boat used for the synthesis. We note that the afterglow performance of phosphors synthesized using different corundum boats does not show much difference (Supplementary Fig. 14), suggesting good reproducibility. Rietveld refinement based on a two-phase model was performed using the general structure analysis system (GSAS) software package^34^. Assuming the Pr at the Y site and the O and F occupying the same Wyckoff site, the Rietveld refinement of the data immediately converged to *R*_p_ = 4.38% and *R*_wp_ = 5.91% (Fig. 2c, Table S1). The site occupancy factor for the F and O atoms was also refined and was determined to be 0.956(3), resulting in a chemical formula of Cs_2_NaY(Pr)F(O)_5.736_□_0.264_, where □ represents anion vacancies. We stress that the existence of a trace amount of Gd^3+^ in the phosphor, owing to the unavoidable contaminant by the precursors (the Y and/or Pr precursors), does not notably affect the afterglow behavior (Supplementary Fig. 15a). The occurrence of a large number of fluorine vacancies can be tentatively attributed to the replacement of F^−^ by O^2−^ that forces the release of F^−^ to satisfy the charge neutrality. We also stress that the Pr^4+^ ions are absent in both as-synthesized and charged products, as suggested by the XANES and electron spin resonance (ESR) spectroscopy, respectively (Fig. 2b, Supplementary Fig. 15b). Based on these information, we speculate that the chemical formula of the as-synthesized product can be written as Cs_2_NaY_0.99_Pr_0.01_F_5.472_O_0.264_□_0.264_. We point out that this corresponds to a molar ratio of oxygen to fluorine of 13.0% when considering the existence of YAG in the product (Table S1), which is comparable to that by the EDS measurement. Collectively, the structural analysis clearly justifies the existence of anion vacancies in the product, which are thus conceived to act as electron traps.

**Fig. 3:**
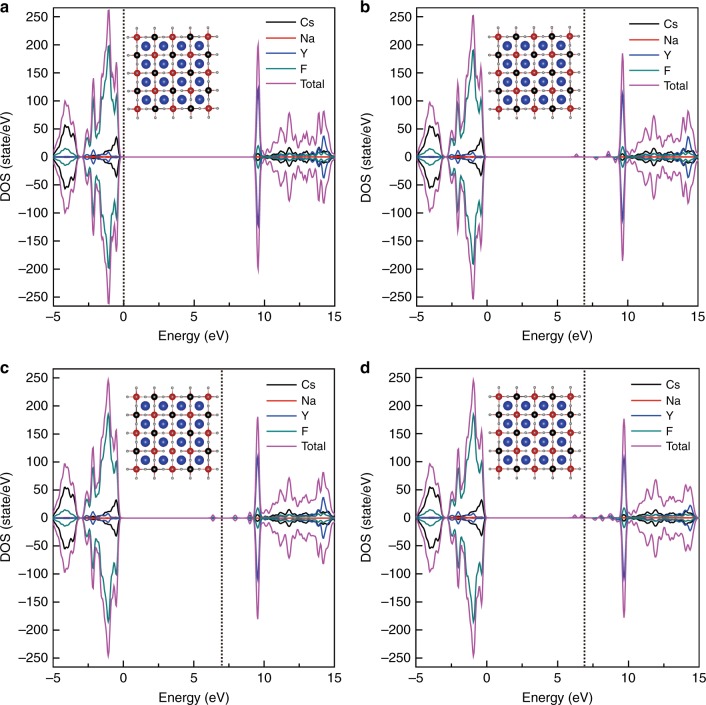
DOS of pristine and defective Cs_2_NaYF_6_. **a** DOS of pristine Cs_2_NaYF_6_. **b** DOS of Cs_2_NaYF_6_ with one fluorine vacancy at the apical site of the [YF_6_] octahedron. **c** DOS of Cs_2_NaYF_6_ with two fluorine vacancies at two apical sites of one [YF_6_] octahedron. **d** DOS of Cs_2_NaYF_6_ with two fluorine vacancies at one apical and one equatorial site of the [YF_6_] octahedron. The insets in (**a**–**d**) show the structures for the calculations, where blue, black, red, and pale spheres represent Cs, Na, Y, and F ions, respectively. The vertical dotted lines in (**a**–**d**) represent the Fermi level. The zero-energy point was set to the Fermi level of pristine Cs_2_NaYF_6_. The Fermi levels of the defect-bearing systems were corrected by aligning the average electrostatic potential (*V*_av_) of F atoms located far from the defects to the *V*_av_ of the same elements in the pristine Cs_2_NaYF_6_

To ascertain the possibility of anion vacancy-mediated trapping of electrons in our product, we performed density functional theory (DFT) calculations. We underscore that there are many possibilities for defects or defect complexes in this type of mixed-anion system with vacancies. To simplify the discussion, we primarily focused on the effect of fluorine vacancies on the electronic structure of Cs_2_NaYF_6_. We first considered three models for defective Cs_2_NaYF_6_ featuring a single fluorine vacancy at the apical site of the [YF_6_] octahedron and two fluorine vacancies at two apical sites or at one apical and one equatorial site of the [YF_6_] octahedron. We note that the calculated band gap for the pristine Cs_2_NaYF_6_ is 9.67 eV, which is comparable to the experimental value^33^. The calculated density of states (DOS) for both pristine and defective Cs_2_NaYF_6_ is shown in Fig. 3a–d. Interestingly, we find that the creation of one fluorine vacancy at the apical site of the octahedron introduces four in-gap states, at approximately 0.18, 0.60, 1.45, and 2.86 eV below the conduction band minimum (CBM) (Fig. 3b). Similarly, creating two fluorine vacancies at two apical sites of one [YF_6_] octahedron gives rise to three in-gap states, at approximately 0.24, 1.26, and 2.85 eV below the CBM (Fig. 3c). By contrast, the formation of two fluorine vacancies at one apical and one equatorial site of the octahedron leads to more complex in-gap states, with the deepest defect level being 3.11 eV below the CBM (Fig. 3d). We also calculated the DOS of the defective Cs_2_NaYF_6_ with two fluorine vacancies at two apical sites or at one apical and one equatorial site of the [NaF_6_] octahedron, and we found similar in-gap states (Supplementary Fig. 16). All these results unambiguously indicate that the introduction of fluorine vacancies in Cs_2_NaYF_6_ results in the appearance of a series of in-gap defect levels with different depths with respect to the CBM. We stress that the deeper traps with>1.3 eV below the CBM predicted here can be supported well by the photostimulated luminescence as shown in Fig. 1e–g.

**Fig. 4:**
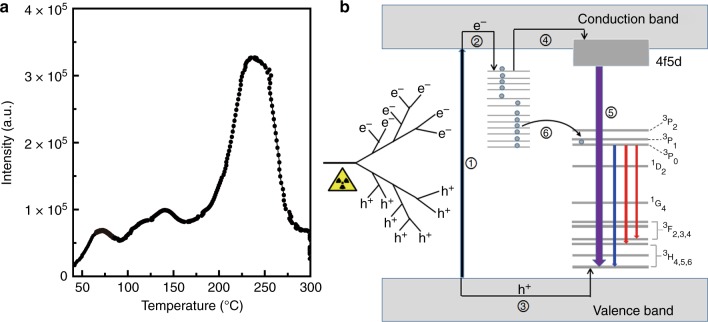
Thermoluminescence spectrum and schematic illustration of the proposed afterglow mechanism. **a** The thermoluminescence spectrum of phosphors with a nominal composition of Cs_2_NaY_0.99_F_6_:0.01Pr^3+^. The sample was irradiated for 1000 s at room temperature, and then was left for 48 h before the thermoluminescence measurement. **b** Proposed afterglow mechanism. The purple, blue, and red lines represent the optical transitions corresponding to the UVC, blue, and red emissions, respectively. Note that the emission corresponding to the transition of 4*f*5*d* → ^3^H_5_ is partially in the UVC spectral range

The existence of fluorine vacancy-related defect levels in our product was also experimentally confirmed by the thermoluminescence measurements. Figure 4a displays the thermoluminescence curve of the persistent phosphor at 48 h after stopping the X-ray irradiation. The trap depths (*E*) relative to the CBM can be calculated by the expression *E* = 0.002*T*_m_, in which *T*_m_ is the temperature for which the thermoluminescence peak is the maximum (in kelvin, K)^2^. Three shallow traps, with respect to those>1.3 eV below the CBM, were observed to have the activation energy values of 0.69, 0.83, and 1.02 eV at 70, 141, and 238 °C, respectively. We note that the determined shallow defects have good consistency with the results calculated by DFT (Fig. 3b–d), although other structural defects, beyond the cases in our DFT calculations (e.g., the formation of [YF_3_] or [NaF_3_] due to the loss of three fluorine in one octahedron or the formation of defect complex consisting of [YF_5_], [NaF_5_] or others), probably also contribute to the formation of these shallow traps.

On the basis of all these results, we posit that the defects associated with fluorine vacancies could act as electron trapping centers with diverse depths with respect to the CBM, making fluoride elpasolite an excellent X-ray-excitable UVC long-lasting phosphor. The use of X-ray as the excitation source signifies that the dominant mechanism for the persistent luminescence observed is by the direct recombination of released electrons with Pr^3+^ ions. Nevertheless, the fact that the visible persistent luminescence decays more slowly than the UVC, both of which are due to the electronic transitions of Pr^3+^, indicates that other processes should also involve the electron detrapping. In view of the existence of the YAG phase in the product which may influence the afterglow, we further synthesized the sample using a platinum crucible; the XRD result suggests the absence of any impurity in the product (Supplementary Fig. 17a). Interestingly, we note that this phase-pure sample shows similar visible PL, but different photoluminescence excitation spectra with respect to the YAG-containing phosphor (Supplementary Fig. 17b,c and Supplementary Fig. 4b). It is well known that the absorption wavelength of ^3^H_4_ → 4*f*5*d* of Pr^3+^ in YAG is longer than that in the elpasolite^33,35^, suggesting that the UVC emissions observed in the YAG-containing phosphor originate from the elpasolite phase and that the observed photoluminescence excitation band in Supplementary Fig. 4 mainly originates from the YAG phase. Additionally, we find that, at 5 min after stopping the X-ray irradiation, the relative intensity of the UVC to the visible emissions for the YAG-containing sample is smaller than that for the phase-pure product (Supplementary Fig. 17d), which signifies that the YAG phase, along with the elpasolite phase, contributes to the visible afterglow. We underscore that, similar to the YAG-containing sample, the UVC afterglow decays faster than the visible cousin in the phase-pure sample (Supplementary Fig. 17e). The decay curve corresponding to the visible emission was further plotted as a function of reciprocal persistent luminescence intensity (*I*^−1^) versus time (*t*) (Supplementary Fig. 17f). The *I*^−1^ ~ *t* at the 50–120 min period for the red persistent luminescence can be fitted linearly, suggesting that a tunneling-related process occurs^11^. Based on all these observations, we propose a plausible mechanism for the UVC and visible persistent luminescence, as schematized in Fig. 4b. Upon X-ray irradiation, the absorption of an X-ray photon yields an energetic, ionized free electron. This hot electron collides with atoms in the material, and it triggers the cascading production of additional ionized electrons^36^. Lower-energy collisions may cause the excitation of valence band electrons into the conduction band, leading to the creation of many electron–hole pairs (process 1 in Fig. 4b). The excited electrons and created holes are subsequently captured by electron traps and Pr^3+^ ions, respectively, based on processes 2 and 3. After long-term X-ray irradiation, the traps are filled. After X-ray irradiation ceases, at the beginning, the electrons are primarily released from shallow traps, followed by transfer to the Pr^3+^ ions through the conduction band (process 4). The Pr^3+^ ion with an electron and a hole can be viewed as an excited Pr^3+^, which releases the energy either through the 4*f*5*d*–4*f*^2^ transition or through the 4*f*^2^–4*f*^2^ transition (process 5) that cause the UVC and visible afterglow, respectively. After the depletion of electrons captured in shallow traps, those in the deep traps can migrate directly to nearby Pr^3+^ ions by tunneling and then they are captured by the 4*f*^2^ energy levels of Pr^3+^, resulting in the visible emissions in the absence of the UVC emission (process 6). We point out that overexposing the phosphor under X-rays leads to weaker UVC emission (Supplementary Fig. 7). This may be caused by some X-ray-induced defects^37^, which remains an open question for further study. We stress that after releasing most of the stored electrons, the phosphor can be recharged, showing a nearly identical afterglow behavior (Supplementary Fig. 18).

## Discussion

Here, we present the discovery of UVC persistent luminescence in a defective, Pr^3+^-doped fluoride elpasolite, and we demonstrate that the development of UVC persistent phosphors is not an insurmountable goal. Specifically, we have found that incorporating oxygen into the lattice results in the formation of a large number of anion vacancies that can serve as electron traps. The extension of the spectral range of persistent luminescence from visible and NIR to UVC opens up a diversity of potential applications.

**Fig. 5:**
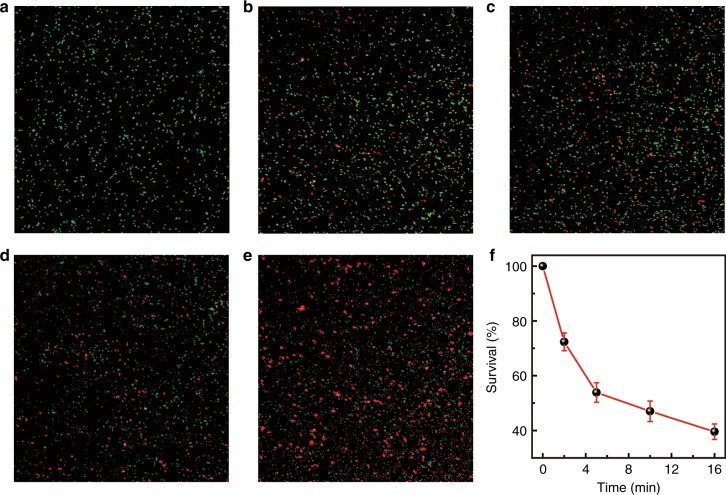
Inactivation of *P. aeruginosa* PAO1 using UVC-afterglow phosphor sheets. **a** Confocal micrograph of the control sample exposed at ambient conditions. **b**–**e** Confocal micrographs of *P. aeruginosa* PAO1 undergoing different irradiation of UVC afterglow. Four phosphor sheets that were irradiated by X-ray for (**b**) 2 min, (**c**) 5 min, (**d**) 10 min, and (**e**) 16 min were used to inactivate *P. aeruginosa* PAO1. The live and dead cells show green and red colors, respectively. **f** The dependence of *P. aeruginosa* PAO1 survival ratios on the X-ray irradiation time of the given phosphor sheets. 100% viability can be maintained when keeping *P. aeruginosa* PAO1 under ambient conditions. The error bars represent the standard error and are obtained based on three independent experiments

As is well known, *Pseudomonas* (*P.*) *aeruginosa* PAO1 is a common Gram-negative and monoflagellated bacterium that can survive in a diversity of environment conditions^38^. The organism can cause disease not only in animals and plants but also in humans. As a proof of concept, we show that the UVC persistent phosphor developed here can be used for killing *P. aeruginosa* PAO1. As shown in Fig. 5a, 100% viability can be maintained when keeping *P. aeruginosa* PAO1 under ambient conditions (i.e., room light, normal atmosphere) for 30 min. We used a total of four sheets of UVC persistent phosphors that were irradiated by X-rays for 2, 5, 10, and 16 min, respectively. The intensity of the persistent luminescence increases with the increasing irradiation time. We note that before irradiation, each sheet was fixed on a u-shaped bracket. At 2 s after the cease of irradiation, the long-persistent luminescent sheet was kept close to the culture dish. Interestingly, we observe that the survival of *P. aeruginosa* PAO1 is associated with the X-ray irradiation time of the sheet, and around 39.6% viability can be maintained for *P. aeruginosa* PAO1 in the dish with the sheet after 16 min of irradiation (Fig. 5b–f). This observation provides direct evidence that the persistent luminescence from our UVC phosphor can be used for sterilization. Thanks to the outstanding penetrating ability of X-rays, the charging of our phosphors are barely influenced by biological tissues (Supplementary Fig. 19), thus highlighting its enormous potential for in vivo applications.

## Conclusions

In summary, we have developed a new class of phosphor that exhibits strong and long-lasting UVC afterglow. A combination of experimental and theoretical results leads us to propose that the structural defects in the elpasolite that are associated with oxygen introduction-induced anion vacancies serve as electron traps, which render it capable of capturing and storing a large number of electrons as triggered by X-ray irradiation. Interestingly, the afterglow intensity of this phosphor is sufficiently strong for sterilization. We believe that the concept shown here may be applicable to other Pr^3+^-doped wide-bandgap compounds, suggesting a series of UVC persistent phosphors with excellent performance. Our finding of this UVC afterglow opens up a new frontier in persistent phosphors, and offers an opportunity for novel applications, such as sterilization, disinfection, drug release, the in vivo killing of cancer cells, anti-counterfeiting, and beyond.

## Materials and methods

### Synthesis of UVC persistent phosphors

Pr-doped polycrystalline fluoride elpasolite phosphors, with nominal compositions of Cs_2_NaY_(1*−x*)_F_6_:*x*Pr^3+^, were prepared by a solid-state reaction method. Cs_2_CO_3_ (1.6290 g, 99.99%, Aladdin, Shanghai, China), NaHCO_3_ (0.4200 g, 99.99%, Aladdin, Shanghai, China), Y_2_O_3_ (0.5588 g, 99.99%, Aladdin, Shanghai, China), NH_4_F (2.2222 g, 99.99%, Aladdin, Shanghai, China), and Pr_6_O_11_ (0.0085 g, 99.996%, Alfa, United States) powders were mixed together with 3 mL of acetone and then ground thoroughly. The obtained powders were thermally treated at 150 °C in air for 7 h, followed by regrinding to obtain a fine powder. The mixture was first sintered at 450 °C for 30 min in air atmosphere. The obtained powders were then reground, followed by sintering at 700 °C for 10 h under a nitrogen atmosphere. The white powders were collected and stored for further characterizations. Corundum boats with a purity of 99% and a platinum crucible were used as vessels for the above synthesis.

### Charging of persistent phosphors

The X-ray irradiation of the product was performed using a calibrated RS-2000 biological irradiator equipped with a tungsten target (160 kV, 25 mA), and the X-ray dose was tuned by changing the irradiation time. The wavelength of X-ray from the irradiator is 0.2106 Å.

### Structural and morphological characterizations

XRD patterns were taken using an X’Pert-Pro MPD diffractometer (Netherlands PANalytical) with a Cu Kα X-ray source (*λ* = 1.540598 Å). TEM images and TEM-EDS mapping were taken with an FEI Tecnai G2 F20 S-TWIN TMP microscope (200 kV). SEM image and SEM-EDS mapping were taken with a Zeiss scanning electron microscope (Zeiss Supra55). We note that three different regions with a size of ~5 μm × 5 μm for the samples were used for the EDS measurement; the O/F ratio was obtained by averaging these data, and determined to be (12.3 ± 2.0)%. XPS was performed on a Rigaku XPS-7000 spectrometer. The carbon peak at 284.6 eV was used as a reference to correct the charging effect.

### Luminescence, afterglow, and thermoluminescence characterizations

Luminescence spectra were recorded by a spectrofluorometer (FLS980, Edinburgh Instruments Ltd.) equipped with a photomultiplier (R928P with an applied voltage of 950 V, Hamamatsu). The persistent luminescence spectra were taken at different time intervals after ceasing the X-ray irradiation. All data regarding the afterglow intensity versus time were recorded from 5 min after stopping the X-ray irradiation. Note that the slit widths of the detection monochromator used for the luminescence and afterglow measurements are 3 nm and 10 nm, respectively, which results in relatively broad afterglow emission bands with respect to photoluminescence bands (Figs. 1a and S4a). Thermoluminescence measurements were performed with a thermoluminescent dosimeter (FJ-427A1), with a heating rate of 1 °C/s from room temperature to 300 °C. The sample was irradiated for 1000 s at room temperature, and then it was left for 48 h before the thermoluminescence measurement.

### Real-time afterglow measurements by a UVC imager

A homemade visible-blind UVC imager was used for this measurement. We note that the sensitive range of this imager is 240–280 nm, which was achieved by adding a bandpass filter. The UVC signals from the samples, which were recorded as the number of photons, were recorded by the UVC imager. To avoid the saturation of photon counts, a relatively low applied voltage was used for recording the initial UVC images. The afterglow decay curves shown in Figs. S10b, c and S11 were drawn based on these measurements. The photostimulated luminescence was monitored by this UVC imager under the excitation of laser diodes with emissions peaking at 450, 730, and 793 nm. To monitor the UVC signal under heating, the sample was heated by a hot plate set to 200 °C and the UVC signal was measured by the UVC imager; note that the first image was taken at 5 s after putting the sample on the hot plate. The distance between the imager and the sample was 70 cm. Both photostimulated and thermostimulated UVC images were taken at 24 h after ceasing X-ray irradiation (the irradiation time: 1000 s). We emphasize that the sensitivity of the UVC imager used is poorer than that of photomultiplier used for afterglow measurements as shown in Fig. 1c.

### Estimation of the power density of the UVC afterglow

We roughly evaluate the power density of the UVC afterglow using a Thorlabs PM200 power meter equipped a power sensor (S120VC, Thorlabs). The detailed measurement method is shown in Supplementary Fig. 20. After considering all possible factors that impact the measurement, the initial afterglow power density at the sample position was roughly estimated to be ca. 14.9 mW/m^2^.

### Synchrotron X-ray measurement

We took the synchrotron XRD measurement using the BL02B2 beam line of SPring-8 to obtain high-quality diffraction patterns at 296 K. The sample was sealed into Hilgenberg glass capillaries with an inner diameter of 0.1 mm, and during the measurement, the capillary was continuously rotating. The X-ray wavelength used is 0.413745 Å. Rietveld refinement was preformed against XRD data utilizing the GSAS program^34^. The room temperature Pr L_III_-edge XANES was taken on the 1W1B beam line of the Beijing Synchrotron Radiation Facility with a stored electron energy of 2.5 GeV and average ring currents of 200 mA. A fixed-exit Si (111) double crystal monochromator was used. Pr_6_O_11_ and Pr(NO_3_)_3_·6H_2_O powders were used as reference samples. Data were collected in the fluorescence mode for the studied sample and in the transmission mode for the reference samples. The XAFS data were analyzed using the IFEFFIT software package^39^.

### Bactericidal experiment

The *P. aeruginosa* PAO1 in the culture dishes with the beef extract peptone medium was grown in an incubator at 35 °C. Three days later, the PAO1 population in the culture dishes was ~10^6^ colony-forming units (cfu)/mL. The culture dishes were removed from the incubator and used for the following inactivation experiment. To perform the sterilization experiment, the samples were tableted by a homemade tablet machine. The Cs_2_NaY_0.99_F_6_:0.01Pr^3+^ powders with a mass of 7.4 g were compressed into a sheet sample of 3 cm in diameter and 4 mm in thickness. Each sheet was fixed on a u-shaped bracket, followed by X-ray irradiation for 2, 5, 10, and 16 min. At 2 s after the end of irradiation, the long-persistent luminescent sheet was held close to the culture dish. The distance between the sheet surface and *P. aeruginosa* PAO1 is ~2 mm. After 30 min, the afterglow sheet was removed, and then the PAO1 was diluted with deionized water and centrifuged at 3000 rpm for 20 min. The PAO1 was then dispersed in 20% NaCl solution and used for the following test. To evaluate the cell membrane integrity, a BacLight live/dead bacterial viability kit (L-7012, Molecular Probes) was used, which allows us to differentiate cells with intact (live) membranes from those with damaged (dead) membranes. The stain was then prepared by diluting 3 μL of each component into 1 mL of distilled water, and then it was kept in the dark for 15 min. We note that at least 2000 cells were scored per sample for the analysis. The *P. aeruginosa* PAO1 suspension was imaged using a confocal laser scanning microscope. A water immersion objective lens was used. The *P. aeruginosa* PAO1 suspension images corresponding to the afterglow sheets with X-ray irradiation for 2, 5, 10, and 16 min were compared to confirm the inactivation effect.

### First-principles calculations

DFT calculations are performed using the Vienna Ab initio simulation package (VASP)^40^. We used the Perdew–Berke–Ernzerhof of generalized gradient approximate (GGA) functional for the description of the exchange and correlation energy of the electrons. Because the GGA usually underestimates the band gap of materials, an orbital-dependent potential was used, including an additional Coulomb interaction (Hubbard *U*)^41–44^. The underestimation of the intraband Coulomb interactions was corrected by the Hubbard *U* parameter and the values of *U*_Cs_(*d*) = 8 eV, *U*_Na_(*p*) = 2 eV, *U*_Y_(*d*) = 4 eV, and *U*_F_(*p*) = 10 eV were used. The ionic potential was described by the projector-augmented wave (PAW) pseudopotential. For *k*-point integration within the first Brillouin zone, a 2 × 2 × 3 Monkhorst–Pack grid for a 2 × 2 × 1 super cell was selected. A plane-wave cutoff energy of 450 eV was applied to the calculations. The convergence criteria for the maximum force and the total energy were set to 0.01 eV Å^−1^ and 1.0 × 10^−4^ eV/atom, respectively. Based on the static states mentioned above, the DOS of pristine and defective Cs_2_NaYF_6_ was calculated.

## Electronic supplementary material

Supplementary materials
