# Supplementary material for: X-ray-activated long persistent phosphors featuring strong UVC afterglow emissions
Source: Light Sci Appl. 2018 Nov 14;7:88. doi: 10.1038/s41377-018-0089-7 (PMC6234205; doi:10.1038/s41377-018-0089-7)
Supplement: Supplementary file 1 — Supplementary materials [file 41377_2018_89_MOESM1_ESM.doc]

Supporting Information

**X-ray-activated long persistent phosphors featuring strong UVC afterglow emissions**

Yan-Min Yang,^1#^ Zhi-Yong Li,^2#^ Jun-Ying Zhang,^3#^ Yue Lu,^3^ Shao-Qiang Guo,^3^ Qing Zhao,^4^ Xin Wang,^1^ Zi-Jun Yong,^2^ Hong Li,^2^ Ju-Ping Ma,^2^ Yoshihiro Kuroiwa,^4^ Chikako Moriyoshi,^4^ Li-Li Hu,^5^ Li-Yan Zhang,^5^ Li-Rong Zheng,^6^ and Hong-Tao Sun^2,*^

^1^College of Physics Science and Technology, Hebei University, Baoding, 071002, China.

^2^College of Chemistry, Chemical Engineering and Materials Science, Soochow University, Jiangsu, 215123, China.

^3^Department of Physics, Beihang University, Beijing, 100191, China.

^4^Department of Physical Science, Hiroshima University, Higashihiroshima, Hiroshima, 739-8526, Japan.

^5^Shanghai Institute of Optics and Fine Mechanics, Chinese Academy of Sciences, Shanghai 201800, China.

^6^Beijing Synchrotron Radiation Facility, Institute of High Energy Physics, Chinese Academy of Sciences, Beijing, 100049, China.

^#^These authors contributed equally.

*To whom correspondence should be addressed.

E-mail: timothyhsun@gmail.com

**
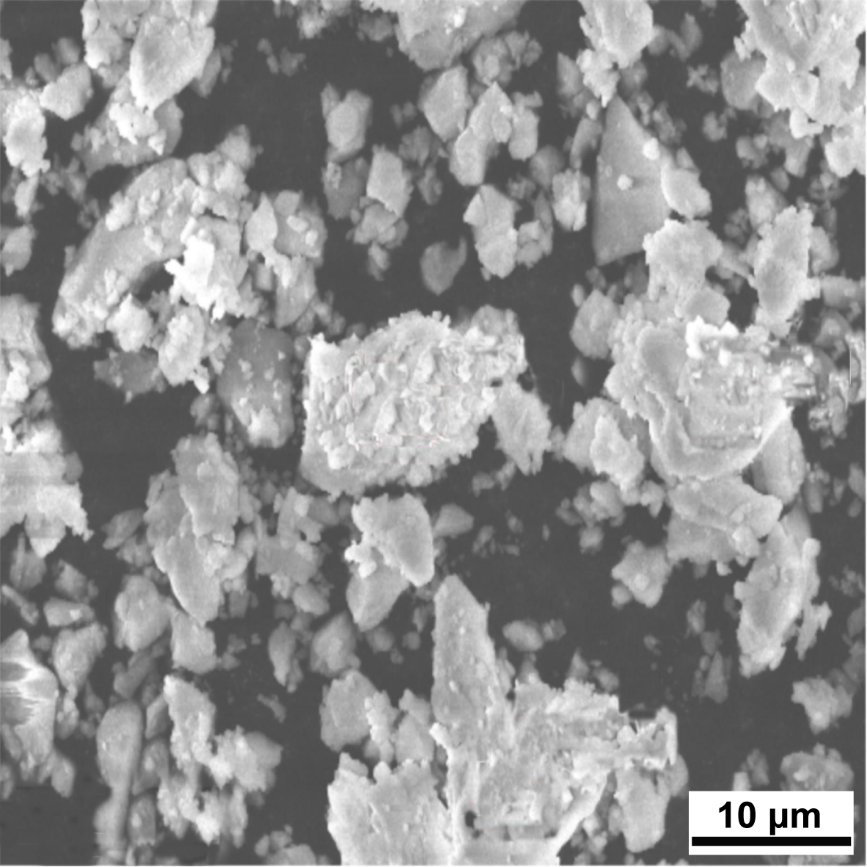
**

**Figure S1.** SEM image of the phosphors with a nominal compositon of Cs_2_NaY_0.99_F_6_: 0.01Pr^3+^.


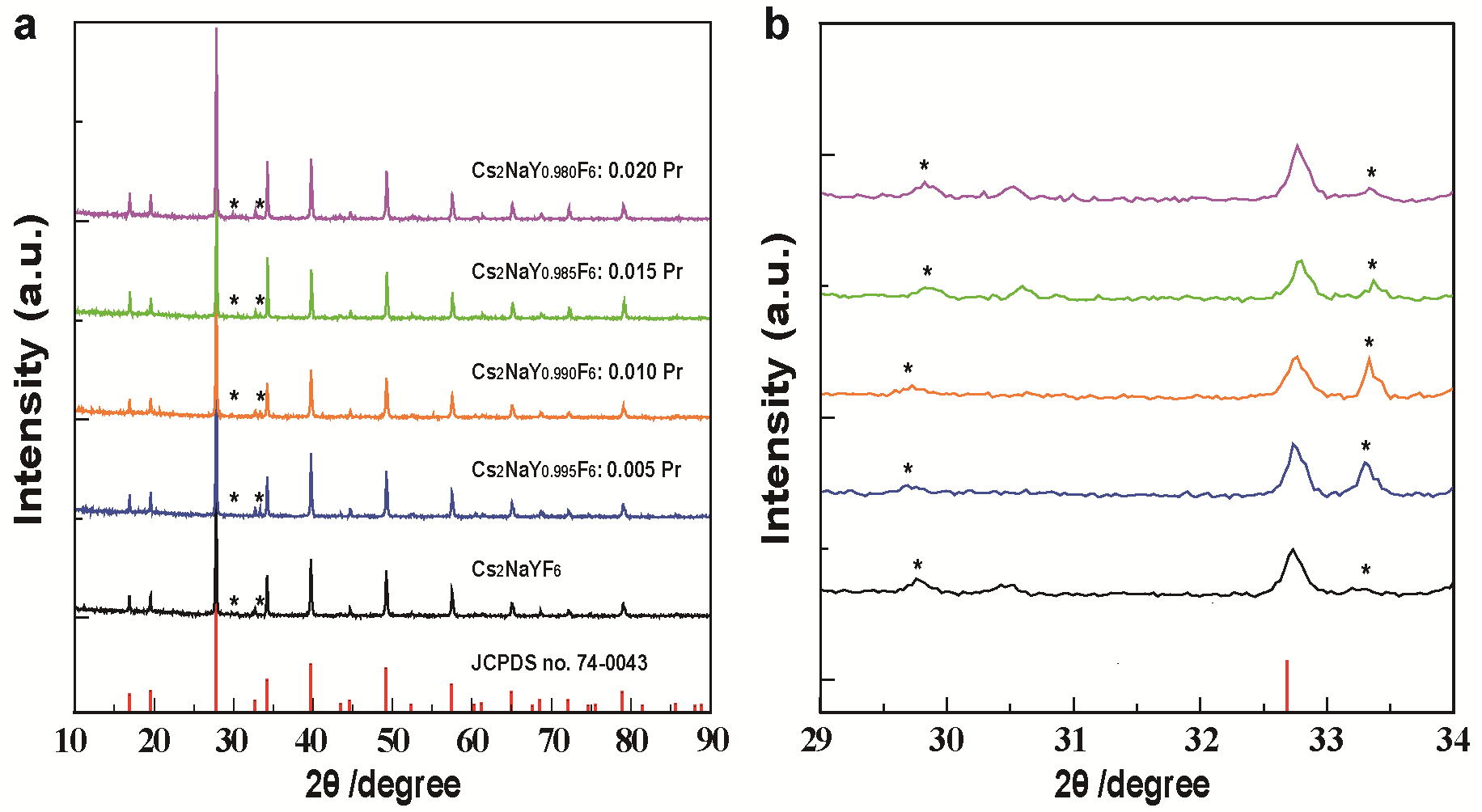


**Figure S2.** a) XRD patterns of undoped and Pr-doped Cs_2_NaYF_6_. The peak denoted by the asterisk is ascribed to the Y_3_Al_5_O_12_ (YAG) phase (JCPDS No. 72-1315). b) Enlarged image of XRD patterns. It is shown that all products contain the YAG phase.


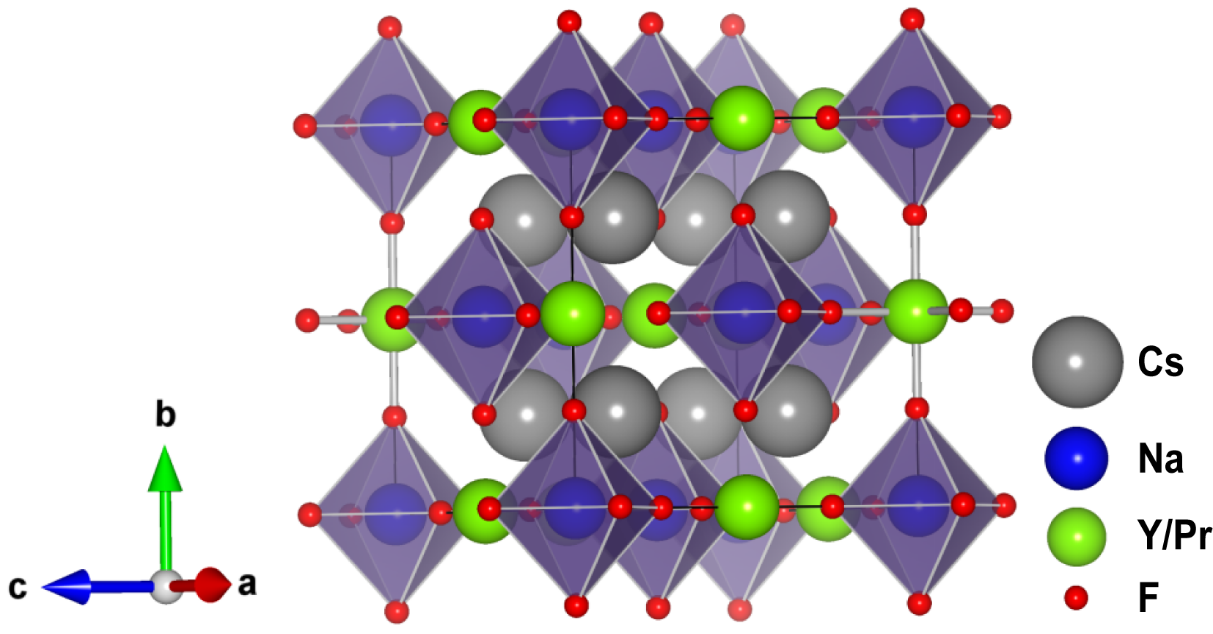


**Figure S3.** Structure of Pr-doped Cs_2_NaYF_6_ crystal.

**
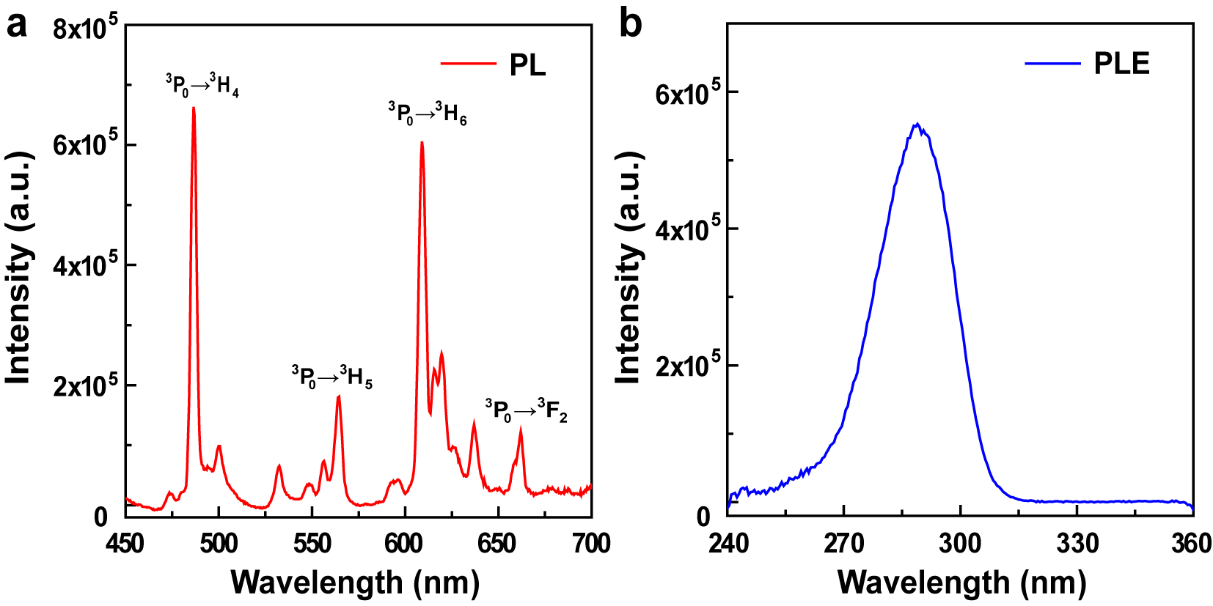
**

**Figure S4.** a) PL and b) PL excitation spectra of the Cs_2_NaY_0.99_F_6_:0.01Pr^3+^ sample. The excitation and detection wavelengths for PL and PLE are 288 nm and 486 nm, respectively. We note that the slit width used here is narrower than that used for the afterglow measurement.


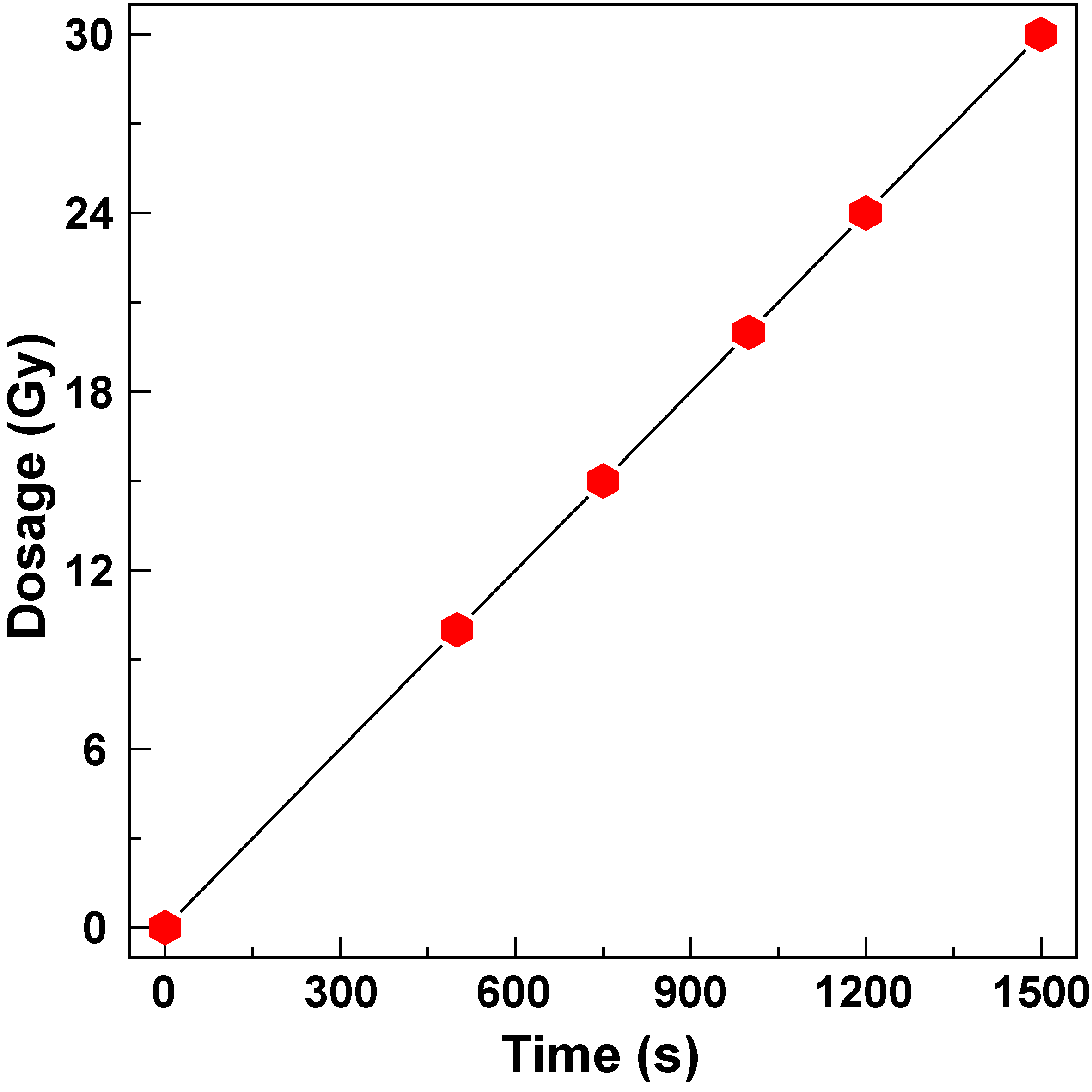


**Figure S5.** Dependence of doses on X-ray radiation time.


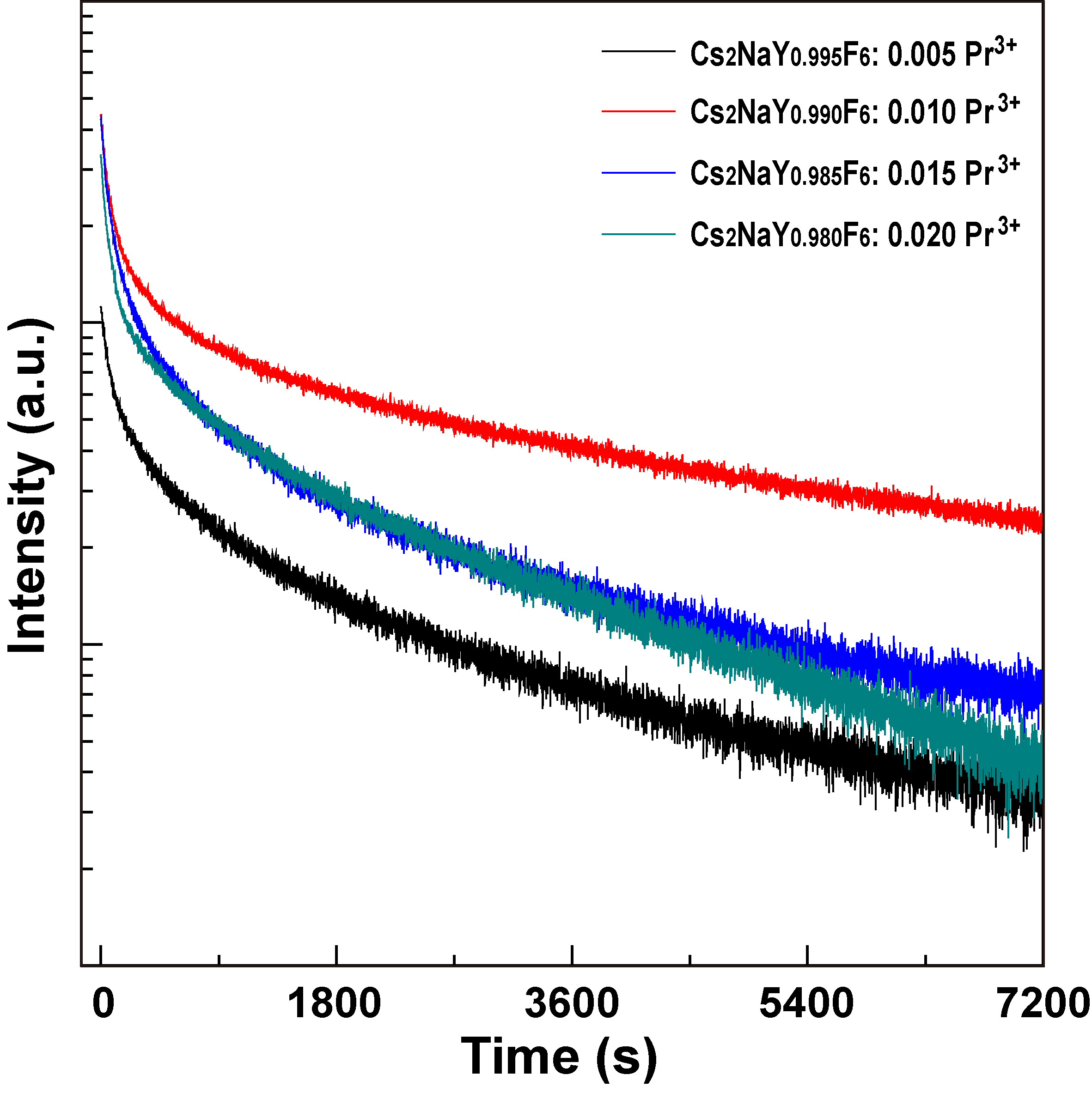


**Figure S6.** Afterglow intensity monitored at 250 nm as a function of time for Cs_2_NaY*_(1-x)_*F_6_:*x*Pr^3+^.


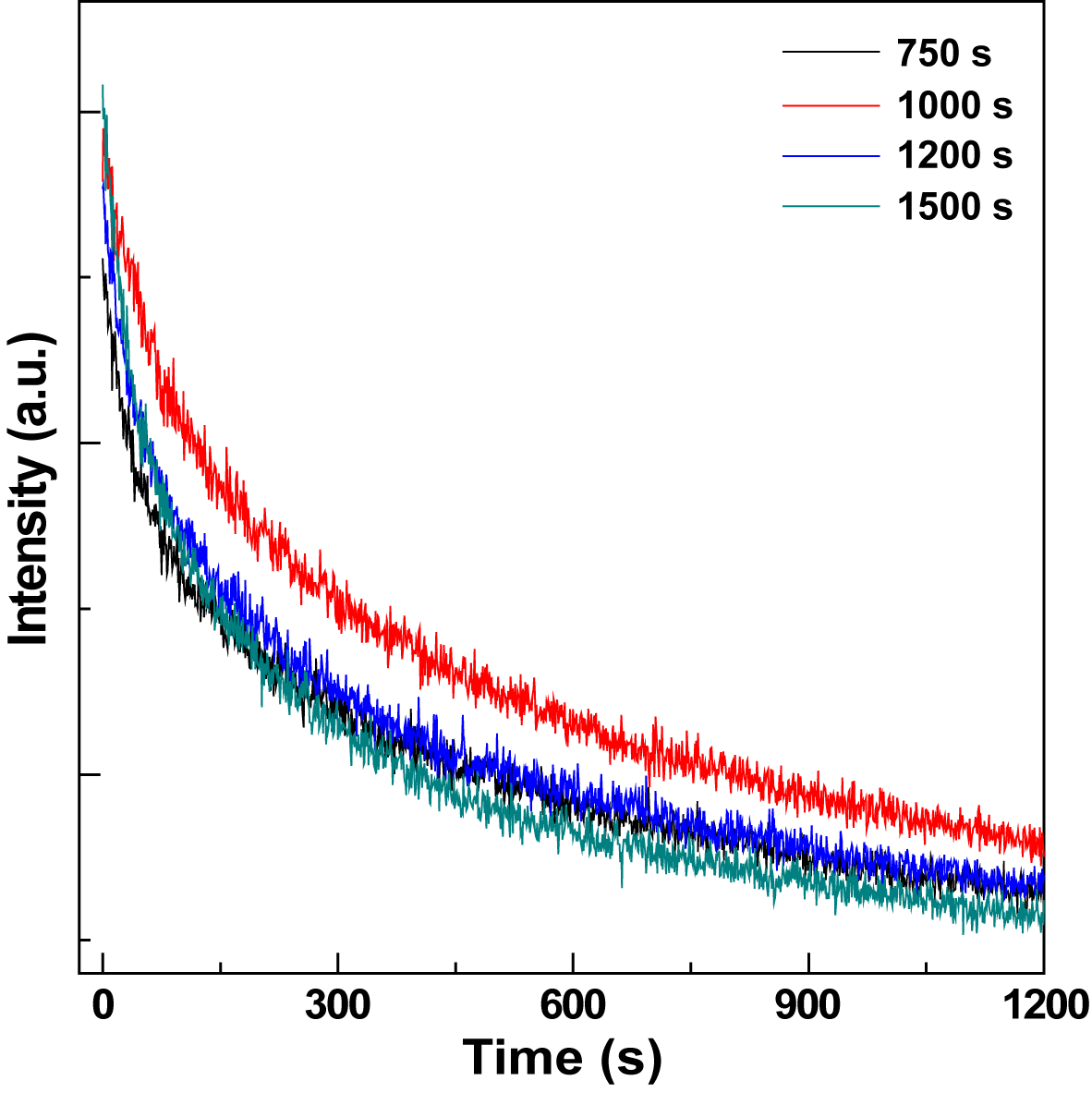


**Figure S7.** Afterglow intensity monitored at 250 nm for Cs_2_NaY_0.99_F_6_:0.01Pr^3+^ irradiated by X-ray for different time.


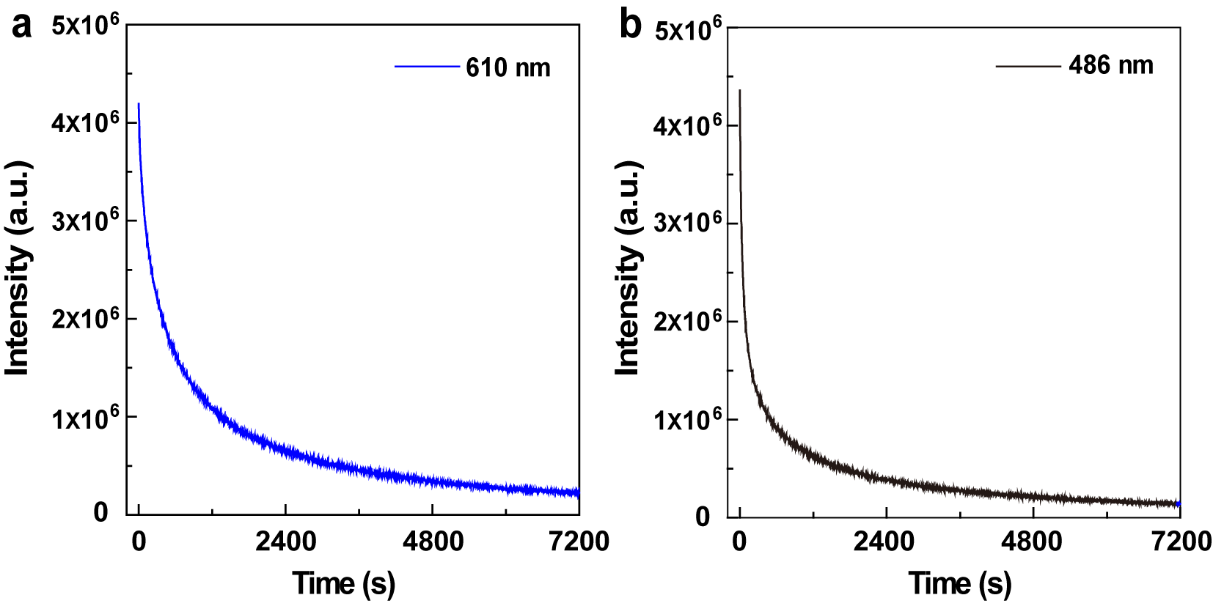


**Figure S8.** Afterglow intensity monitored at (a) 610 nm and (b) 486 nm for Cs_2_NaY_0.99_F_6_:0.01Pr^3+^ irradiated by X-ray for 1000 s.


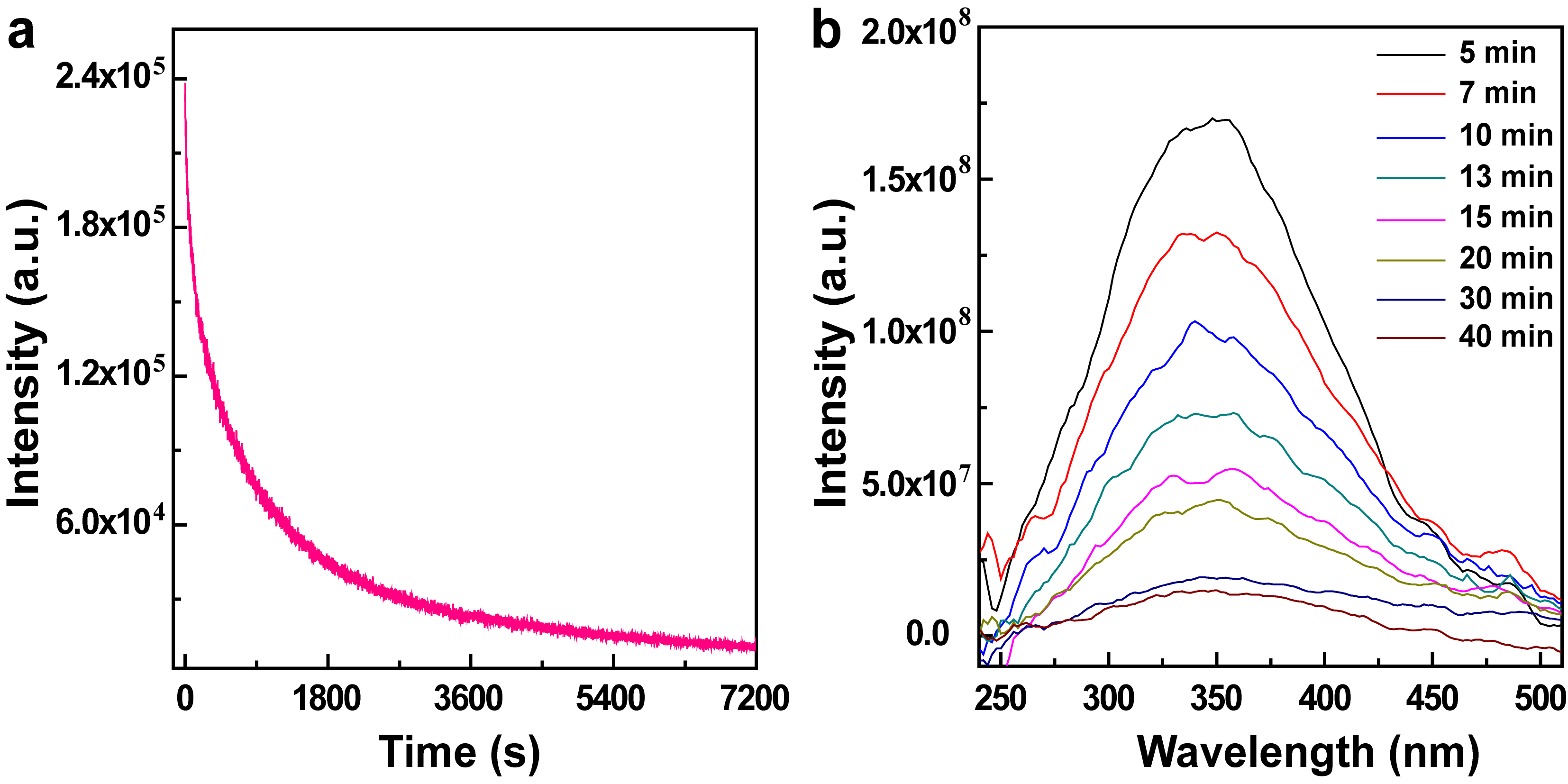


**Figure S9.** a) Afterglow intensity monitored at 345 nm for the sample with a nominal composition of Cs_2_NaYF_6_. The sample was irradiated by X-ray for 1000 s. b) Afterglow spectra recorded at different time after the stoppage of X-ray irradiation.


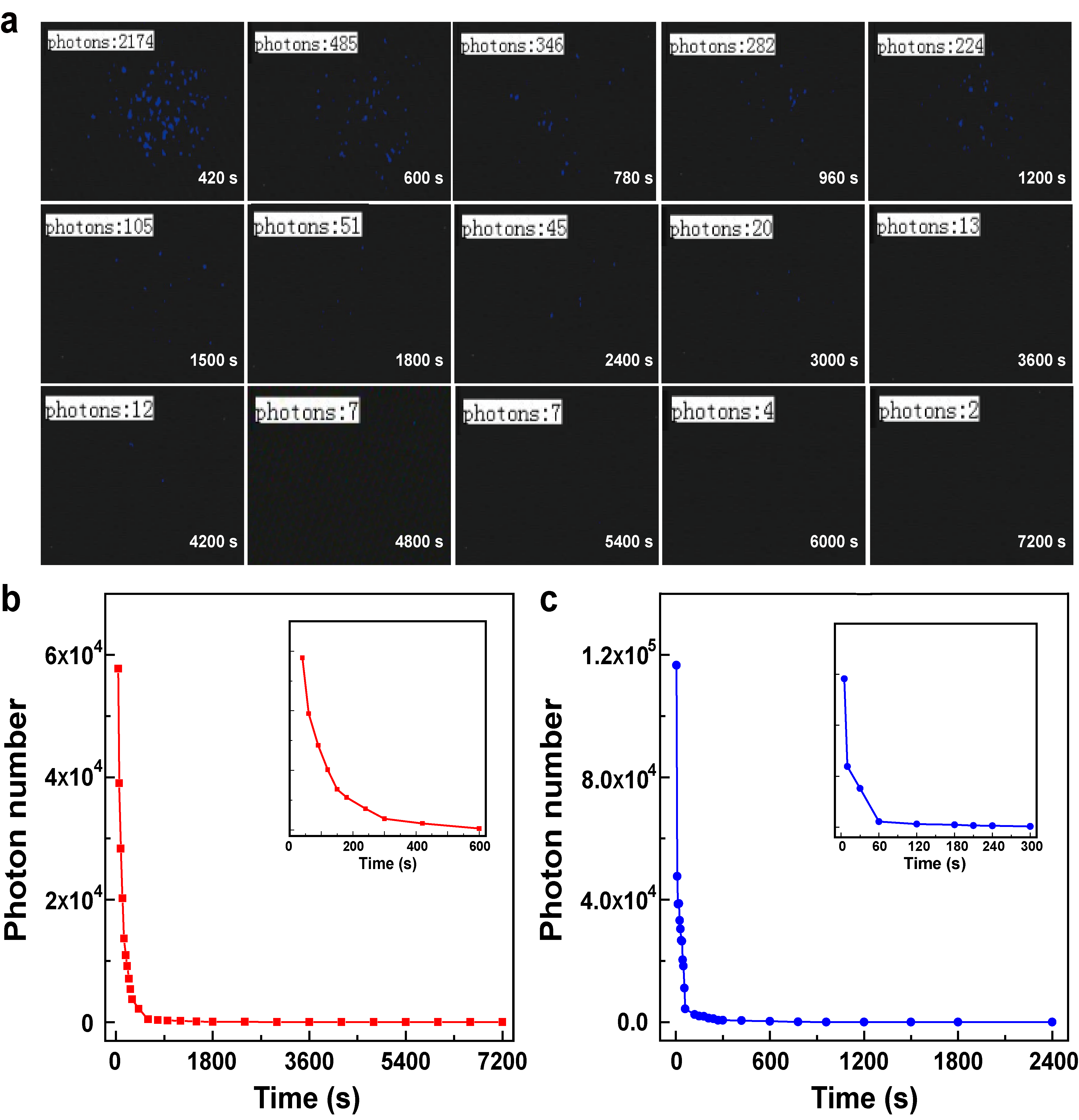


**Figure S10.** a) Additional UVC images of phosphors taken after 300 s for Cs_2_NaY_0.99_F_6_:0.01Pr^3+^. The photon number is indicated in each image. We note that due to the poor sensitivity of the UVC imager used, the photon number taken after 3600 s is less than 13, approaching the noise level of the imager. However, the PMT used for the afterglow measurement in Fig. 1b is more sensitive than the UVC imager, thus allowing the detection of UVC signals after 3600 s. b) UVC photon number of Cs_2_NaY_0.99_F_6_: 0.01Pr^3+^ irradiated by X-ray for 1000 s. Inset shows the enlarged image. The recording of photon number begins from 40 s after the X-ray irradiation, because it is necessary to transfer the irradiated sample from the chamber of an irradiator to the measurement setup of a UVC imager. c) UVC photon number of the 24-h-decayed Cs_2_NaY_0.99_F_6_:0.01Pr^3+^ when heated at 200 °C on a hot plate. The recording begins from 5 s after putting the sample on the hot plate.


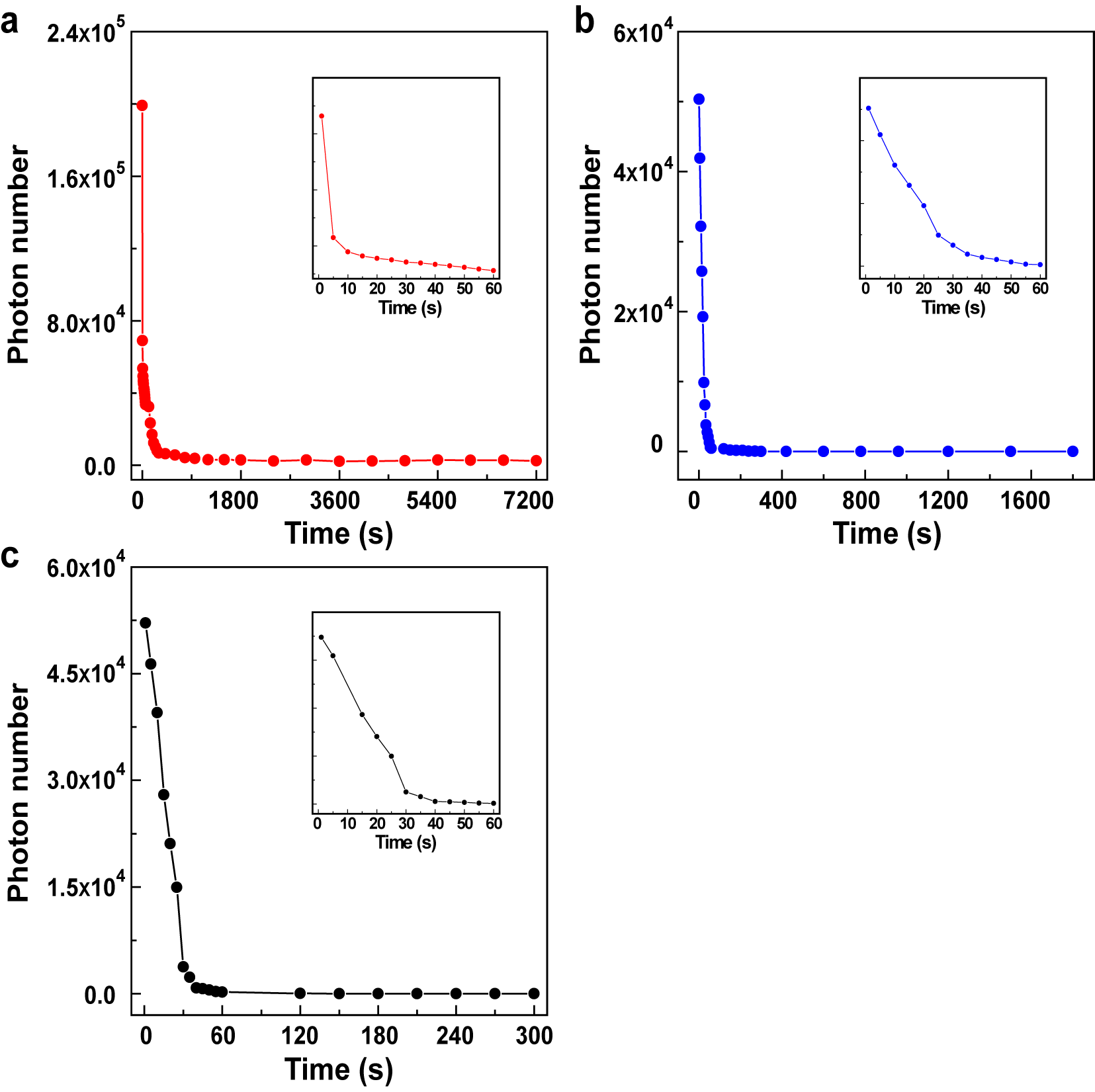


**Figure S11.** UVC photon numbers of the 24-h-decayed phosphors under laser irradiation with different wavelengths of (a) 450 nm, (b) 730 nm, and (c) 793 nm. The excitation power density is 1.77 W/cm^2^ for these excitations. Inset shows the enlarged images.


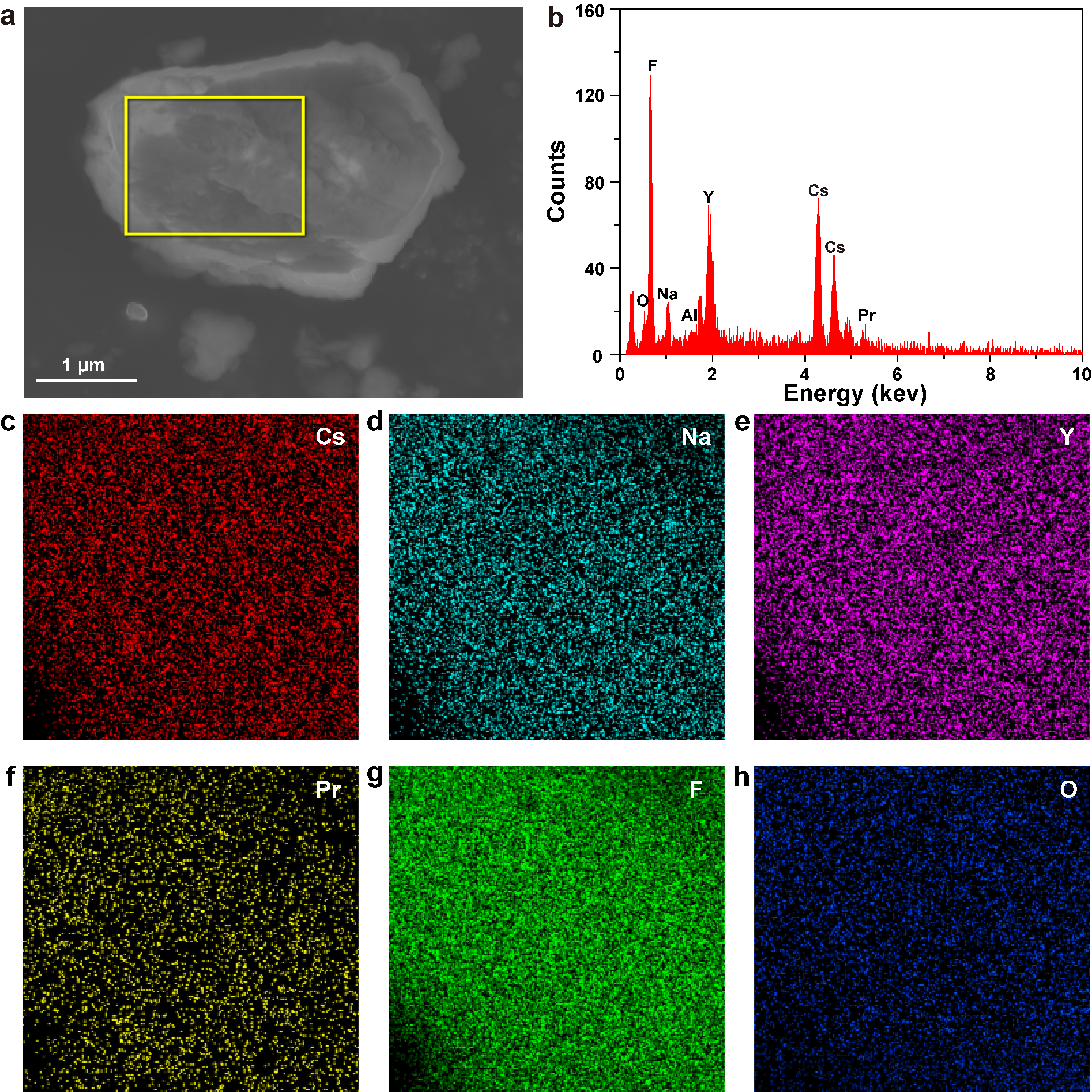


**Figure S12.** a) SEM image of Cs_2_NaY_0.99_F_6_:0.01Pr^3+^. b) EDS spectrum of Cs_2_NaY_0.99_F_6_:0.01Pr^3+^. c-h) EDS mappings of Cs, Na, Y, Pr, F, and O.


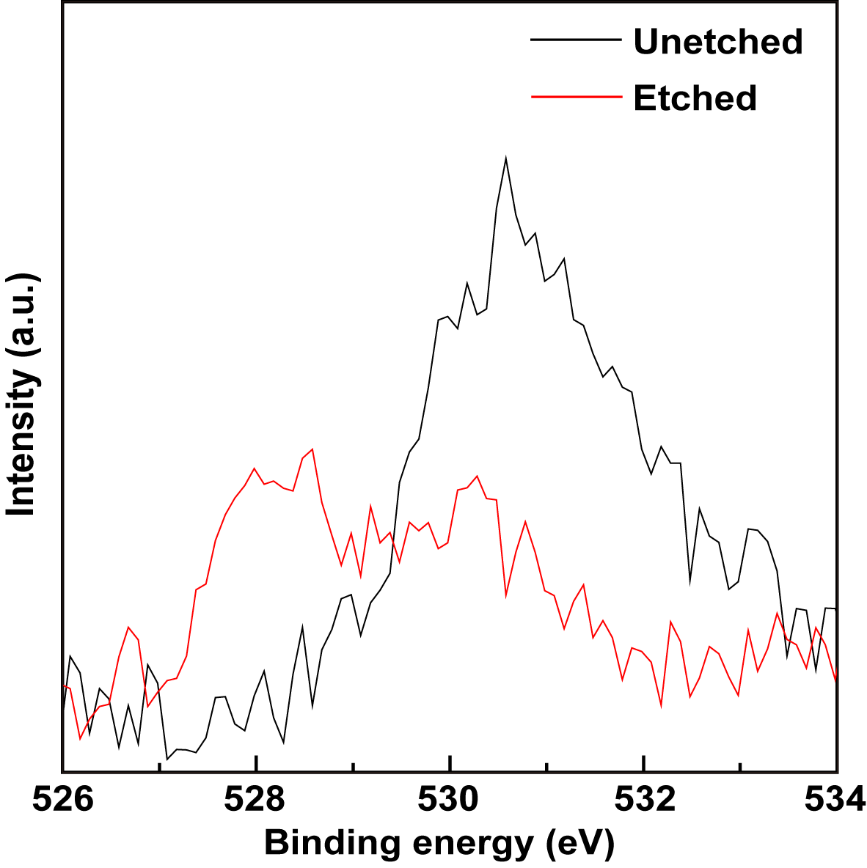


**Figure S13.** O 1s XPS spectra of as-synthesized and argon-plasma-etched Cs_2_NaY_0.99_F_6_:0.01Pr^3+^ powders. The etching time is 60 s. We note that a peak at 530.6 eV is observed for the as-synthesized powder, which can be assigned to hydroxide groups absorbed on the surface.^1^ After plasma etching, two peaks at around 528.3 and 530.2 eV appear, which can be assigned to oxygen bond of Y-O-Na and oxygen in the vicinity of an anionic vacancy (ref. 2), respectively. We note that the absence of notable signal assigned to oxygen bond of Y-O-Na for the unetched sample suggests that the oxygen at the surface does not mainly exist in the form of Y-O-Na, but in the form of hydroxide groups. The XPS result gives a direct indication of the existence of oxygen in the bulk of the sample. Combined with the result of Rietveld refinement, our results suggests the existence of anion vacancies that could be associated with the existence of oxygen that forces the release of F^-^ to satisfy the charge neutrality.


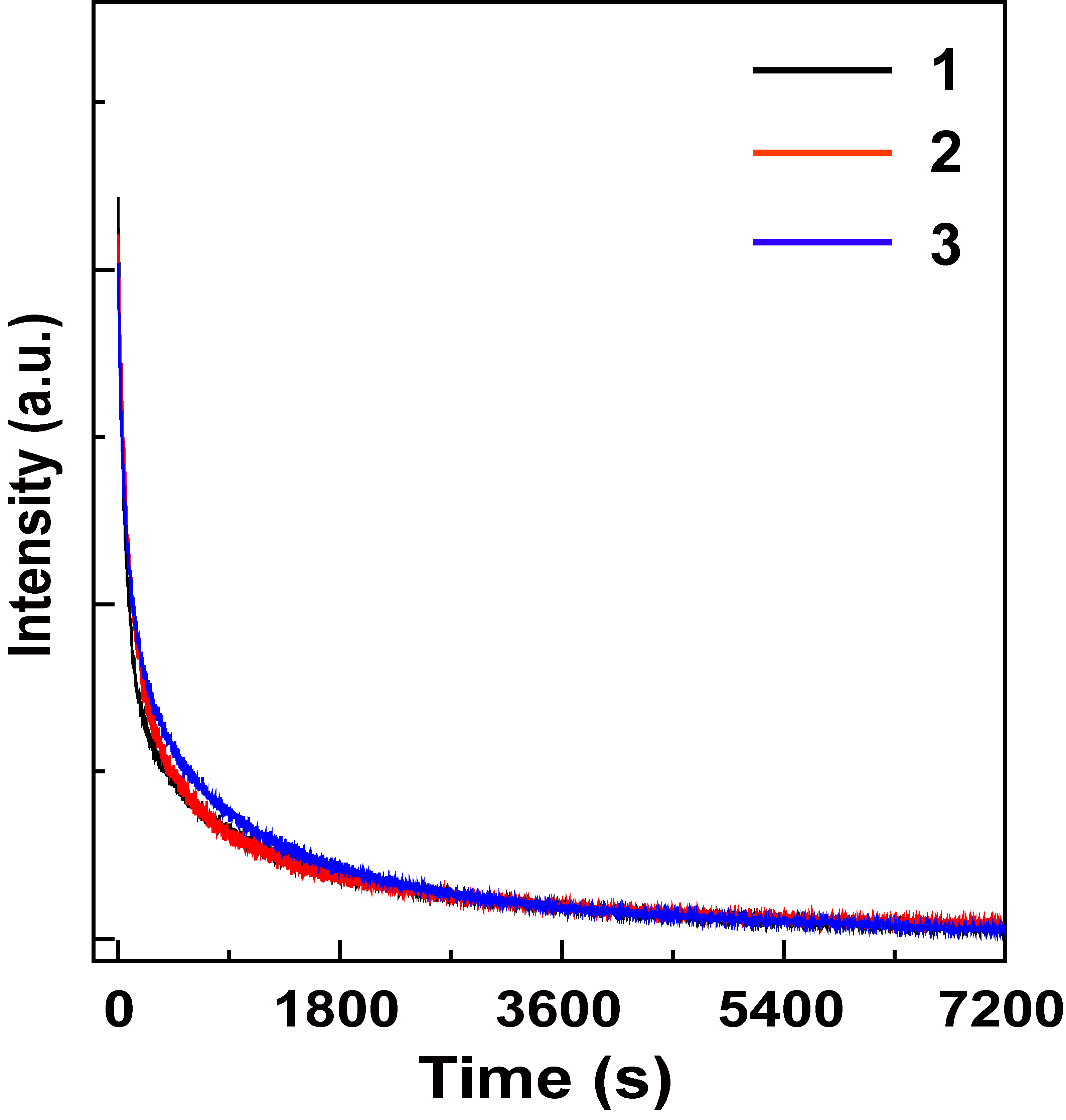


**Figure S14.** Afterglow decays from phosphors synthesized using different corundum crucibles. The afterglow detected at 250 nm from these phosphors does not show much difference.


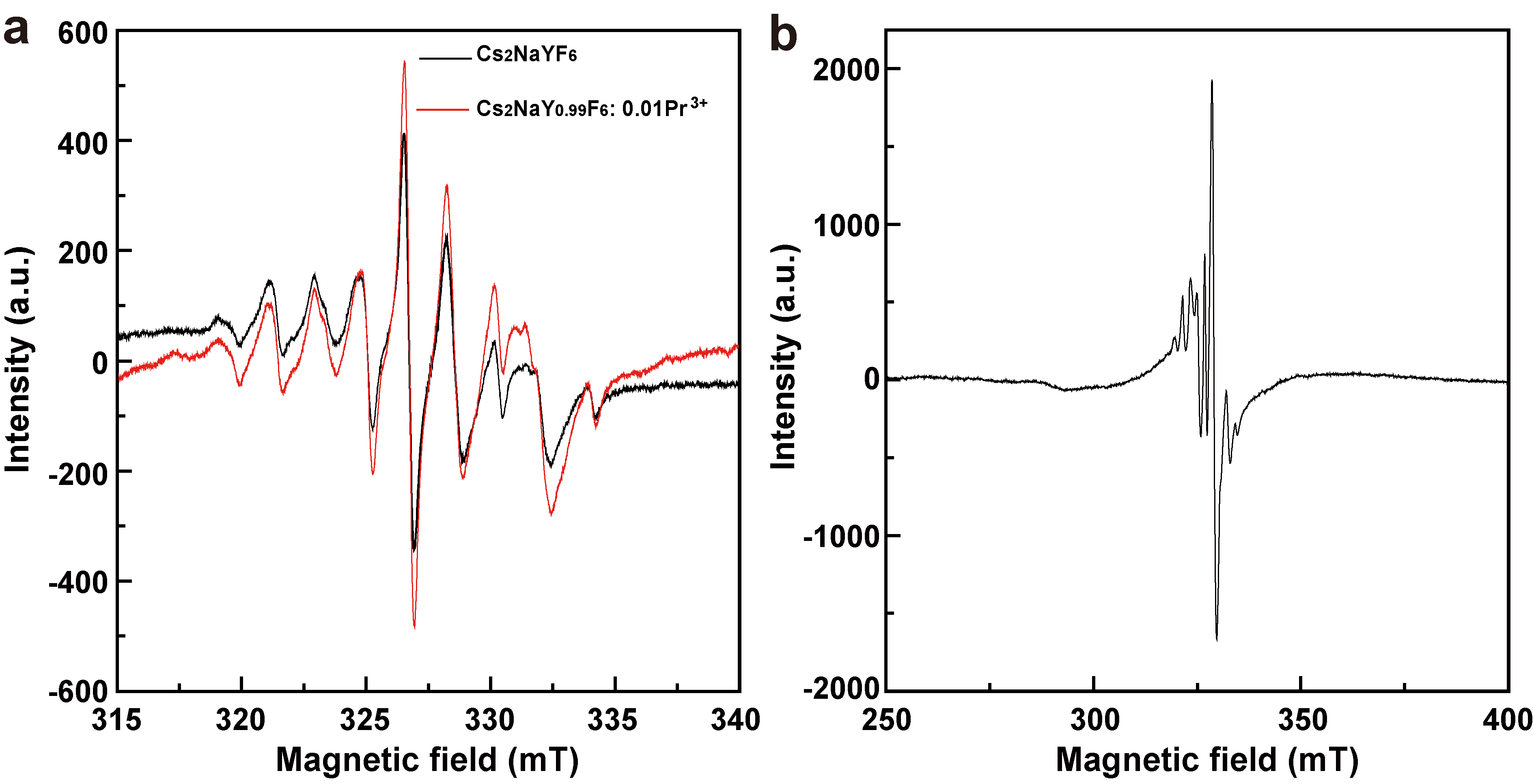


**Figure S15.** a) ESR spectra of Cs_2_NaYF_6_ and Cs_2_NaY_0.99_F_6_:0.01Pr^3+^ taken at room temperature. b) ESR spectrum of Cs_2_NaY_0.99_F_6_:0.01Pr^3+^ taken at 173 K after X-ray irradiation. The observed signal can be assigned to the Gd^3+^ ions that were introduced as unavoidable contaminant by the precursors (Y and/or Pr precursor) ^3^. We note that in our product even after X-ray irradiation, no detectable ESR signal that can be assigned to Pr^4+^ was found^3^. The amounts of both powders used for the ESR measurement were same. The stronger signal of doped powders suggests that some Gd^3+^ ions were introduced by the Pr precursor. The Gd/Y molar ratio in doped powders is determined to be 0.0083% by using inductively coupled plasma mass spectroscopy (iCAPTM Qc, Thermo Scientific).

**
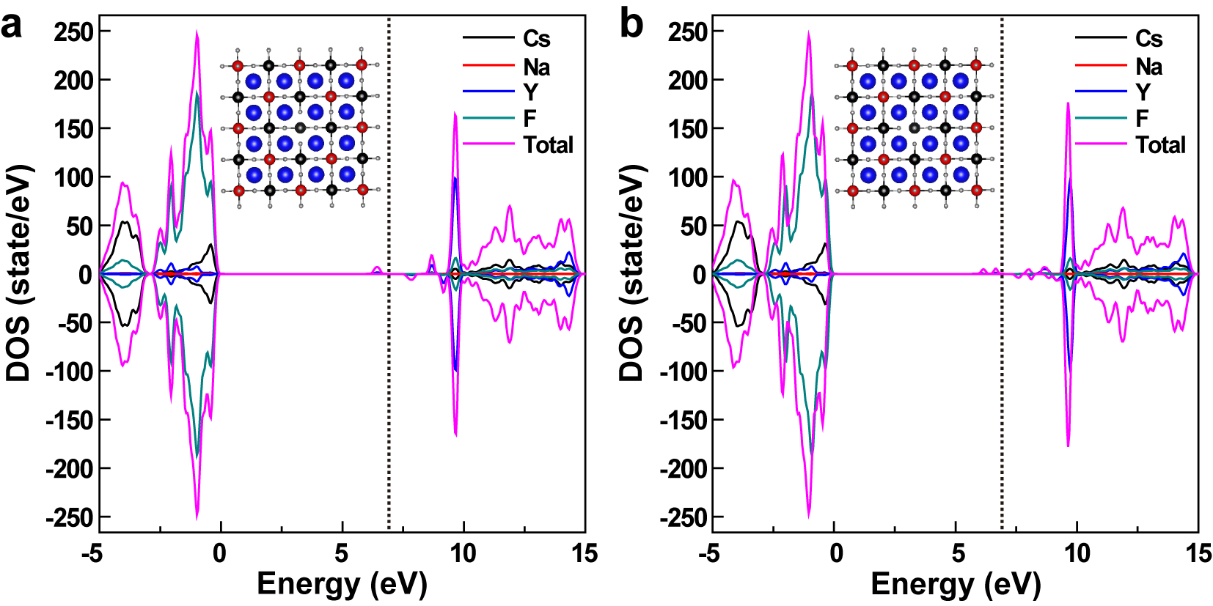
**

**Figure S16.** DOS of defective Cs_2_NaYF_6_. a) DOS of Cs_2_NaYF_6_ with two fluorine vacancies at two apical sites of one [NaF_6_] octahedron. b) DOS of Cs_2_NaYF_6_ with two fluorine vacancies at one apical and one equatorial site of the [NaF_6_] octahedron. Insets in a and b show the structure used for the calculations, where blue, black, red, and pale spheres represent Cs, Na, Y, and F ions, respectively. The vertical dotted lines in (a, b) represent the Fermi level. The Fermi level of the systems with defects were corrected by aligning the average electrostatic potential (V_av_) of F atoms located far from the defects to the V_av_ of the same elements in the pristine Cs_2_NaYF_6_.


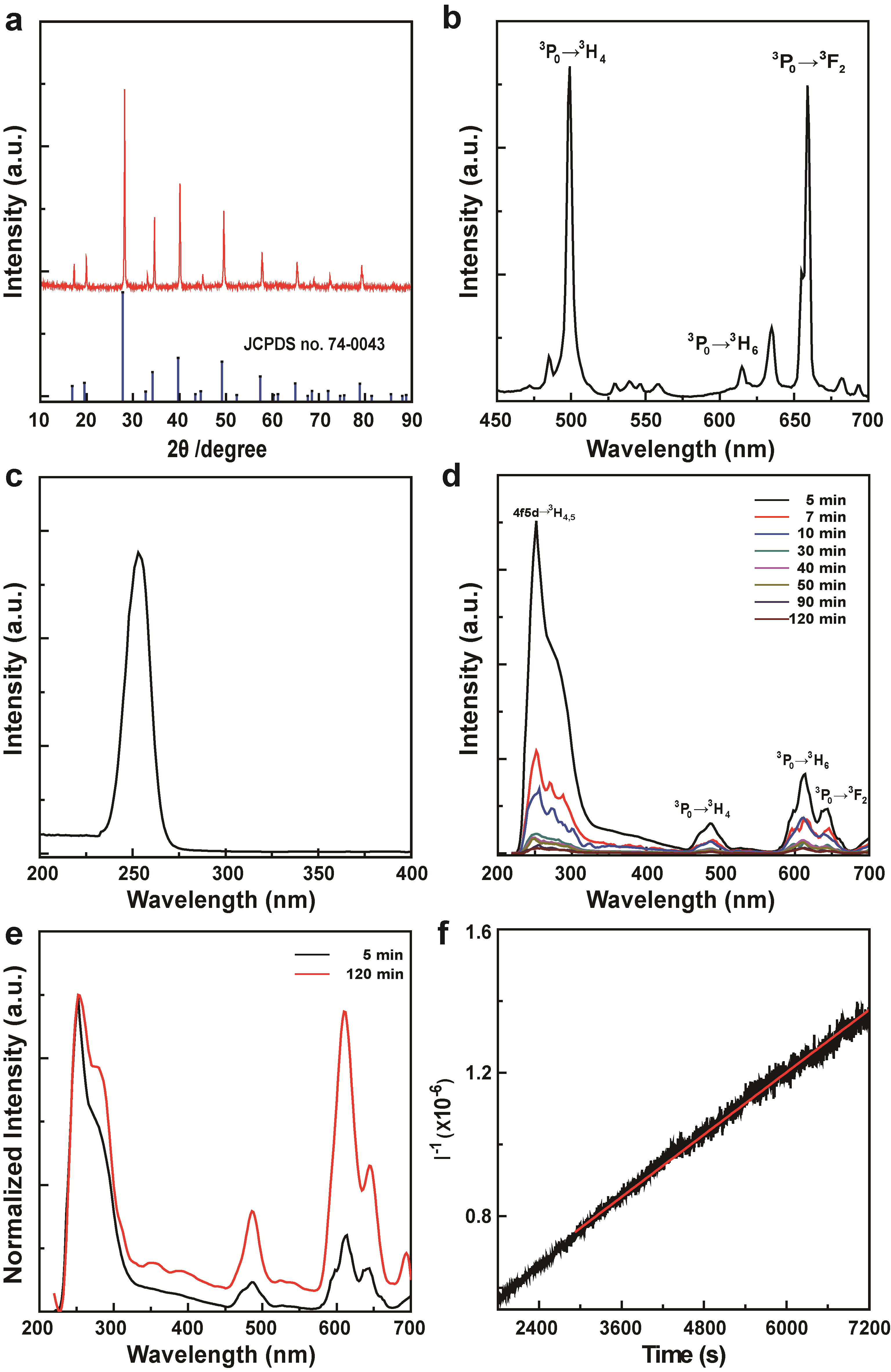


**Figure S17.** Structure and luminescence properties of the sample with a nominal composition of Cs_2_NaY_0.99_F_6_:0.01Pr^3+^ synthesized using a platinum crucible. a) XRD pattern of the product. b) PL and c) PL excitation spectra of the sample. The excitation and detection wavelengths for PL and PLE are 252 nm and 615 nm, respectively. d) Afterglow spectra recorded at different time after ceasing X-ray irradiation. e) Normalized afterglow spectra recorded at 5 min and 120 min after ceasing X-ray irradiation. f) Reciprocal persistent luminescence intensity (*I*^-1^) at 610 nm as a function of time (*t*). The red line is the fitted result.


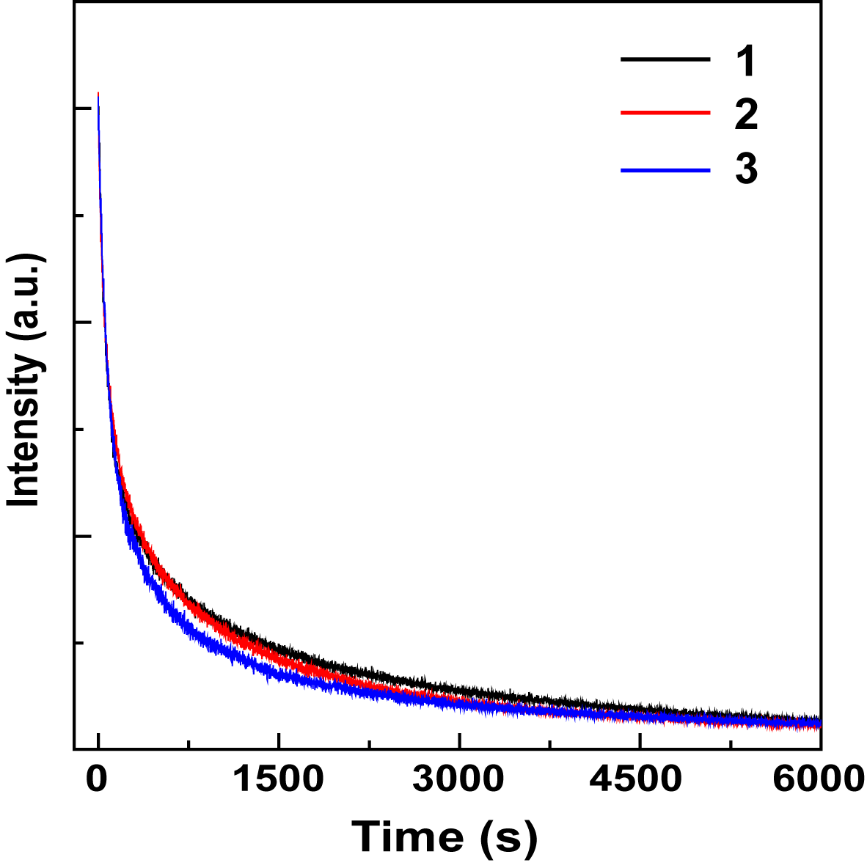


**Figure S18.** Afterglow intensity monitored at 250 nm for Cs_2_NaY_0.99_F_6_: 0.01Pr^3+^ undergoing repeated X-ray irradiation. The curve (1) represents the decay of the sample irradiated by X-ray for 1000 s. After 3 h, the sample was irradiated by X-ray for 1000 s, and then the decay curve (2) was taken. Another three hours later, the sample was irradiated again by X-ray for 1000 s, and then the decay curve (3) was taken. It is clear that the UVC persistent phosphors can be repeatedly charged by X-ray. The little difference of these curves may be due to the occurrence of X-ray-induced structural defects^3^.

**Figure S19.** Afterglow decay curves monitored at 250 nm for Cs_2_NaY_0.99_F_6_: 0.01Pr^3+^ without (black) and with (red) coverage of fresh grade breast with a thickness of 5 cm. The irradiation time by X-ray is 1000 s in both cases. For the red curve, the measurement process is as follows. First, the X-ray penetrates the grade breast to charge the phosphors. After that, the grade breast was removed and the afterglow was measured by a spectrofluorometer (FLS980, Edinburgh Instruments Ltd.).

**
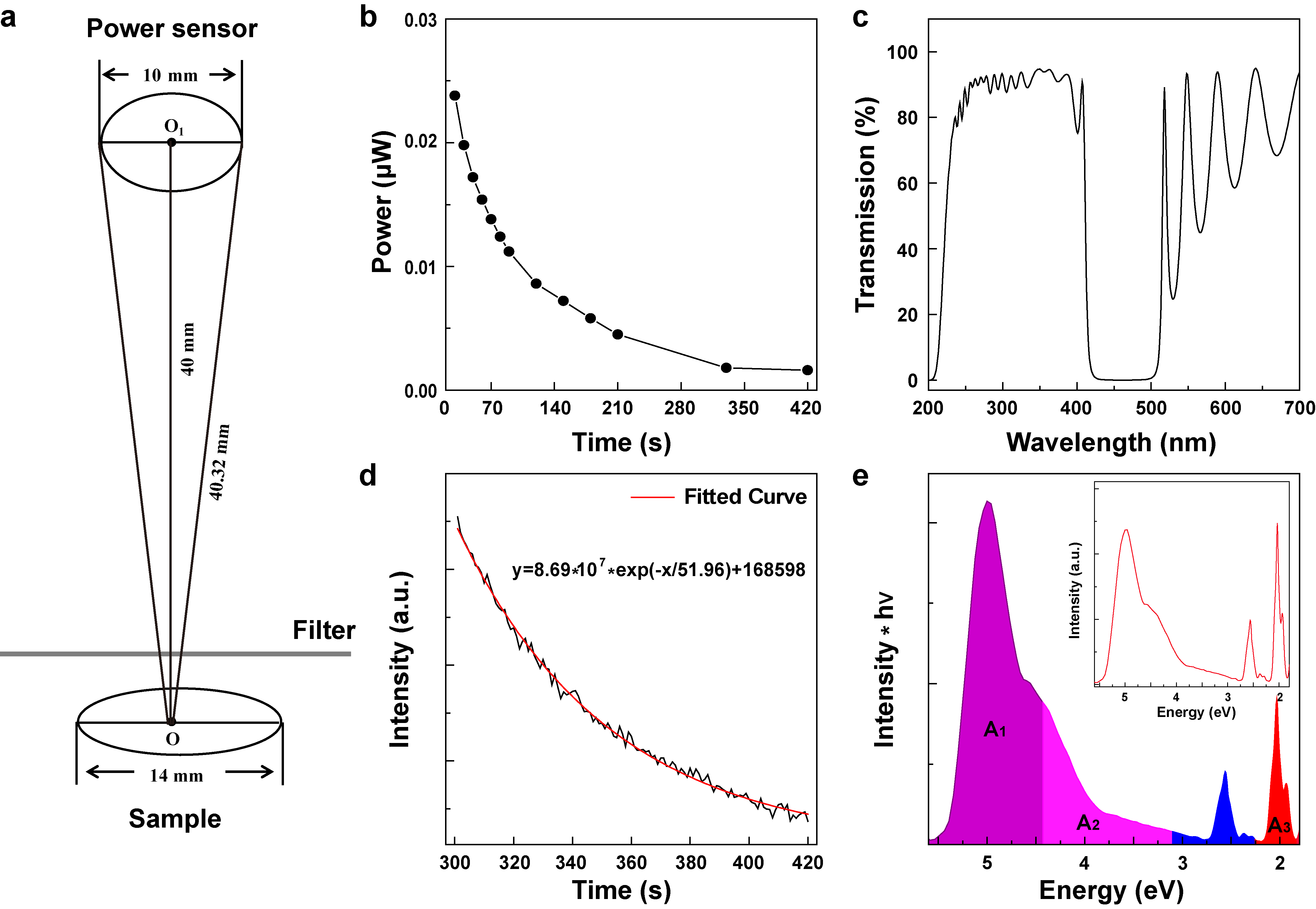
**

**Figure S20.** a) Schematic illustration of the measurement setup for the afterglow power density of Cs_2_NaY_0.99_F_6_:0.01Pr^3+^. The irradiation time by X-ray is 1000 s. The afterglow power was taken from 30 s after stopping the X-ray irradiation. The distance between the sample and the power sensor is 40 mm. The diameters of the phosphor disk and the power sensor are 14 and 10 mm, respectively. b) Measured afterglow power at the position of power sensor as a function of time. It is noted that the power of the noise from the background is ca. 0.0015 μW. c) Transmission spectrum of the optical filter used in (a). The transmissions in the ranges of 220-400 nm and 550-680 nm are around 87% and 61%, respectively. d) Fitted curve based on the early afterglow decay. The decay curve was taken from 5 min after stopping the X-ray irradiation. The fitted result is indicated in the figure. e) Intensity multiplied by the photon energy as a function of the photon energy at 5 min after ceasing X-ray irradiation. This data reflects the optical power distribution of phosphors over the UVC and visible ranges. Considering the transmission spectrum of the filter used, signals from three regions denoted A_1_, A_2_ and A_3_ were detected by the power sensor. Inset shows the afterglow spectrum recorded at 5 min after ceasing X-ray irradiation.

The rough estimation of the initial power density is as follows. The afterglow power is 0.0223 μW at the power sensor position at 30 s after stopping the X-ray irradiation, when subtracting the power of noise from the measured value. Based on the fitted curve of the early afterglow decay as shown in (d), the intensity of afterglow at t=0 s is 1.78 times stronger than that at t=30 s. We thus knew that the power at the position of power sensor is 0.0397 μW at t=0 s. We note that the afterglow in the ranges of 220-280 nm, 280-400 nm and 550-680 nm can partially transmit the filter, as indicated in (c). Combined with the transmissions of the filter at different photon energies with the corresponding areas of three regions in (e), A_1_, A_2_ and A_3_, we can know the ratio of UVC light to all detected signals, from which we can know the power in the UVC at 0 s (P_1_) after stopping X-ray irradiation. *A_1_*, *A_2_* and *A_3_* are the areas of the corresponding A_1_, A_2_ and A_3_ regions, which are 3.546×10^9^, 3.983×10^9^, and 4.999×10^8^, respectively. The calculation details are as follows,

P_1_=0.0397×*A_1_*×87%/[(*A_1_*+*A_2_*)×87%+ *A_3_*×61%]=0.0179 μW

We note that the UVC afterglow decays faster than the visible ones, thus resulting in underestimation of the initial UVC power density when using the afterglow spectrum at 5 min as a reference.

Since the power sensor can only detect partial signals from the phosphor disk, we further estimated the UVC optical power at the position of the disk by assuming a semi-sphere irradiation geometry. Meanwhile, we assume the afterglow from each position in the disk is roughly same with that from the central position (i.e., the “O” position in (a)). We then evaluated the total UVC optical power at the sample position based on the following calcuation,

P= P_1_×2π×40^2^/(π×5^2^)=2.291 μW

Then the power density at the position of the disk was obtained by dividing P by the area of the phosphor disk, which was determined to be 14.9 milliwatts per square meter.

**Table S1.** Structural parameters refined for the sample. The molar ratio of oxygen to fluorine determined by Rietveld refinement is 13.0% when considering the existence of YAG in the final product, which is comparable to that by the EDS measurement.

| Phase | Atoms | *x* | *y* | *z* | Occupancy | U_iso_*100(Å^2^) |
| --- | --- | --- | --- | --- | --- | --- |
| Cs_2_NaY_0.99_Pr_0.01_F_5.472_O_0.264_□_0.264_ | Cs  Na  Y/Pr  F/O | 0.2500  0.5000  0.0000  0.2367(4) | 0.2500  0.5000  0.0000  0.0000 | 0.2500  0.5000  0.0000  0.0000 | 1  1  1  0.956(3) | 2.11(3)  0.67(1)  0.79(2)  0.78(5) |

| Phase | Atoms | *x* | *y* | *z* | Occupancy | U_iso_*100(Å^2^) |
| --- | --- | --- | --- | --- | --- | --- |
| Y_3_Al_5_O_12_ | Y  Al  Al  O | 0.2500  0.0000  0.2500  0.0284(1) | 0.1250  0.0000  0.3750  0.0586(2) | 0.0000  0.0000  0.0000  0.6679(2) | 1  1  1  1 | 0.20(9)  2.73(2)  5.40(9)  1.70(9) |

| Phase | Space group | Cell params | Cell vol | Reliability indexes | Wt. Frac. |
| --- | --- | --- | --- | --- | --- |
| Cs_2_NaY_0.99_Pr_0.01_F_5.472_O_0.264_□_0.264_  Y_3_Al_5_O_12_ | Fm-3m  Ia-3d | a=b=c=9.0615(4) Å  α=β=γ=90°  a=b=c=12.0163(4) Å α=β=γ=90° | 744.05(6)Å^3^  1735.04(9)Å^3^ | R*_wp_* 5.91 %  R*_p_* 4.38 % | 95.62 %  4.38 % |

**References**

## [1] McCafferty E, Wightman J. Determination of the concentration of surface hydroxyl groups on metal oxide films by a quantitative XPS method. *Surf. Interface Anal.* 1998; 26: 549-564.

## [2] Zhang K, Ma CG, Zhang JY, Liu BM, Zhou Y. *et al.* Giant Enhancement of Luminescence from Phosphors through Oxygen-Vacancy-Mediated Chemical Pressure Relaxation. *Adv. Optical Mater.* 2017; 5: 1700448.

## [3] Pawlik T, Spaeth JM. Investigation of Radiation-Induced Defects in Cs_2_NaYF_6_. *Phys. Status Solidi B* 1997; 203: 43-53.
